# How does the updated Nutri-Score discriminate and classify the nutritional quality of foods in a Norwegian setting?

**DOI:** 10.1186/s12966-023-01525-y

**Published:** 2023-10-10

**Authors:** B. Øvrebø, A. L. Brantsæter, K. Lund-Iversen, L. F. Andersen, M. M. Paulsen, M. H. Abel

**Affiliations:** 1 Department of Food Safety, Norwegian Institute of Public Health, Oslo, Norway; 2 Centre for Sustainable Diets, Norwegian Institute of Public Health, Oslo, Norway; 3 Research Administrative Support, Norwegian Institute of Public Health, Oslo, Norway; 4 Institute of Basic Medical Sciences/Department of Nutrition, University of Oslo, Oslo, Norway; 5 Centre for Evaluation of Public Health Measures, Norwegian Institute of Public Health, Oslo, Norway

**Keywords:** Nutrient profiling, Nutrition, Nutri-Score, Food-based dietary guidelines, Front-of-pack nutrition label

## Abstract

**Background:**

The Nutri-Score is a candidate for the harmonized mandatory front-of-pack nutrition label enabling consumers in the European Union to make healthier food choices. Nutri-Score classifies foods (including beverages) from A (high nutritional quality) to E (low nutritional quality) based on the foods’ qualifying and disqualifying components. We aimed to evaluate the updated Nutri-Score for foods (2022) and beverages (2023) in a Norwegian setting by exploring its ability to discriminate the nutritional quality of foods within categories. Additionally, we assessed Nutri-Scores’ ability to classify foods in accordance with the Norwegian food-based dietary guidelines (FBDGs).

**Methods:**

The updated Nutri-Score was calculated for 1,782 foods in a Norwegian food database. The discriminatory ability of the updated Nutri-Score was considered by exploring the distribution of Nutri-Score within categories of foods using boxplots and frequency tables, and by examining which qualifying and disqualifying components that contributed most to the Nutri-Score class. Accordance with the Norwegian FBDGs was assessed by exploring Nutri-Score for foods specifically mentioned in the guidelines.

**Results:**

Overall, the updated Nutri-Score seemed to discriminate the nutritional quality of foods within categories, in a Norwegian setting. The foods’ content of salt and the beverages’ content of sugar were components contributing the most to Nutri-Scores’ discriminatory ability. Furthermore, in most cases the updated Nutri-Score classified foods in accordance with the Norwegian FBDGs. However, there were minor inconsistencies in how Nutri-Score classified certain foods, such as the inabilities to discriminate between full-fat and low-fat/leaner cheeses, cremes and processed meats (sausages), and between whole grain and refined pasta/rice.

**Conclusions:**

We observed an overall acceptable discriminatory performance of the updated Nutri-Score in a Norwegian setting and in most cases the updated Nutri-Score classified foods in accordance with the Norwegian FBDGs. However, minor inconsistencies were observed. Together with the FBDGs, the updated Nutri-Score could be a useful tool in guiding consumers towards healthier food choices in Norway, but consumer evaluations are warranted to fully assess the performance of the updated Nutri-Score in a Norwegian context.

**Supplementary Information:**

The online version contains supplementary material available at 10.1186/s12966-023-01525-y.

## Introduction

Diet is a major contributor to health [1]. Low intake of fruit, vegetables, fish, unsaturated fat, and whole grains, as well as high intake of salt, sugars, and saturated fat, have been linked to suboptimal metabolic risk factors and increased risk of non-communicable diseases and all-cause mortality [1–3]. Improving diet quality through policies are important for both individual and public health.

Front-of-pack nutrition labels (FoPNLs) are of interest because they aim to help consumers make healthier food choices and to stimulate food reformulation [4], thereby improving diet quality. The World Health Organization (WHO) urges governments to implement nutrition labelling policies [5] and FoPNLs have been identified as an important tool [5, 6]. The Farm-to-fork strategy commits the European Commission to propose a harmonized mandatory FoPNL for the European Union by 2023 [7]. Various FoPNLs are proposed and used worldwide and within Europe [4], e.g. warning labels, NutrInform, Multiple Traffic Lights, and Nutri-Score. How the FoPNLs discriminate nutritional quality of foods (indicating the healthiness of foods) depend on their underpinning nutrient profiling algorithm [8]. As the aim of FoPNLs ultimately is to improve diet quality, it is particularly important that the implemented labels align with what constitutes a healthy diet, defined in the national dietary guidelines [4, 5, 9]. The various FoPNLs may not always align with national nutrition policies which can confuse consumers and discredit the FoPNL [10].

Nutri-Score is a debated contender for the harmonized FoPNLs in the European Union [11–13]. Nutri-Score classifies foods and non-alcoholic beverages (hereon covered by the term foods) from A (high nutritional quality) to E (low nutritional quality) based on the foods’ qualifying and disqualifying components relevant for health [14]. Nutri-Score has been shown to identify healthier products, has a high ability to discriminate nutritional quality for food groups [15, 16] and was reported to be consistent with nutritional recommendations across eight European countries [17]. However, researchers [17, 18] and the scientific committee tasked with the revision of Nutri-Score [19] have identified areas for potential improvements, such as better scores for plant-based oils with favorable nutrient composition and for fish and seafood [18]; enhanced discriminatory ability for whole grain products and beverages; and better alignment with recommendations for products with a high content of sugar or salt. For this reason, the Nutri-Score Scientific Committee published proposed updates for the Nutri-Score for foods in 2022 [20] and for beverages in 2023 [21] (together hereon referred to as the updated Nutri-Score). The update covered improvements in many of the aforementioned challenges and was reported to be more in alignment with food-based dietary guidelines (FBDGs) in the European countries engaged in Nutri-Score [20, 22].

To our knowledge, there is currently no published scientific paper presenting an evaluation of the updated Nutri-Score for foods and beverages. More knowledge about how the updated Nutri-Score performs in different countries with different food patterns and dietary guidelines is important for understanding the potential for Nutri-Score to function as a harmonized FoPNL across European countries. As part of the NewTools-project [23], aiming to create more sustainable and healthy food systems, we evaluated the updated Nutri-Score in a Norwegian context. In the present study we first aimed to explore the ability of the updated Nutri-Score to discriminate the nutritional quality of foods within food categories in a Norwegian setting. Secondly, we assessed Nutri-Scores’ ability to classify foods in accordance with the Norwegian FBDGs.

## Methods

### Food composition database

We used the food composition database KBS, version 7.4, AE-22 at the Department of Nutrition, University of Oslo, Norway, in the evaluation of the updated Nutri-Score. The database mainly contains generic foods consumed in Norway and the necessary nutrients for calculation of Nutri-Score, except the foods’ proportion of fruit, vegetables and legumes and presence of non-nutritive sweeteners. The two latter components were estimated as described under Nutri-Score calculation. Of the 4,199 foods in the database, 2,404 were excluded as products were not eligible for the Nutri-Score, such as alcoholic beverages; herbs and spices; meal replacement bars; special dietary foods; baby foods; and home-cooked foods and dishes not representative of products sold in Norwegian stores. Due to missing on total sugar, 13 foods were additionally excluded. We calculated Nutri-Score for whole foods (e.g., fruits and raw meat) and composite foods. The final sample for analyses consisted of 1,782 products.

### Nutri-Score calculation

The updated Nutri-Score comprises three algorithms: one for general foods [20], one for fats, oils, nuts and seeds, and another for beverages [21], presented in Additional file 1. Energy, sugars, fat, saturated fat, salt, protein, fiber (per 100 g) and the proportion of fruit, vegetables and legumes were used to calculate the Nutri-Score. For beverages, the presence/absence of non-nutritive sweeteners was also used.

To estimate the fruit, vegetables and legumes proportion, the component was initially set to 100% for all pure fruit, vegetables and legumes as defined by Nutri-Score [24]. For composite foods, the proportion was automatically calculated based on the recipes in the database. For foods with missing recipes that contained fruit, vegetables and legumes, information from similar or the actual products was used to estimate the proportion if it exceeded 40%, the threshold for points in Nutri-Score [20]. For beverages, presence/absence of non-nutritive sweeteners was determined manually using product descriptions in the database, online ingredient lists or similar products.

In general, the Nutri-Score was calculated by allocating 0 to 20 points for each unfavorable component in a food, while each favorable component provided 0 to 7 points [20, 21]. By subtracting the favorable points from the unfavorable points, each food was given a Nutri-Score total sum of points (hereon total points). Lower and negative total points indicate higher nutritional quality (Table 1). The total points determine the Nutri-Score class, ranging from A-E, using set thresholds (Table 1). The specific components for unfavorable and favorable points vary across the three algorithms and there were specific rules for foods exceeding a certain number of unfavorable points, and for cheese and red meat. For detailed information on the algorithms and calculation of the updated Nutri-Score see Additional file 1 and the “Update report from the Scientific Committee of the Nutri-Score 2022” (pages 130–135) [20] and the “Update of the Nutri-Score algorithm for beverages (2023)” (pages 72–75) [21].

**Table 1:** Nutri-Score total points thresholds for class and color [20, 21]

Nutri-Score total points for general foods	Nutri-Score total points for fats, oils, nuts and seeds	Nutri-Score total points for beverages	Class	Color
≤ 0	≤ -6	Water^a^	A	Dark green
1 to 2	-5 to 2	≤ 2	B	Light green
3 to 10	3 to 10	3 to 6	C	Yellow
11 to 18	11 to 18	7 to 9	D	Light orange
≥ 19	≥ 19	≥ 10	E	Dark orange

^a^Plain water was automatically given Nutri-Score class A

### Food categorization

The included foods were categorized by the authors into main- and subcategories for evaluating the updated Nutri-Score in a Norwegian setting, based on how others categorize foods [25], existing categories in the food database, and food categories specifically mentioned in the Norwegian FBDGs. Beverages and fats, oils, nuts and seeds were in separate categories due to distinct algorithms [20, 21]. The main food categories were predominantly based on raw materials and products thereof with distinct nutritional characteristics, similar to Szabo de Edelenyi et al. 2019 [25], except composite foods difficult to categorize. This resulted in the 13 mutually exclusive main food categories which provided an overall impression of the distribution of Nutri-Score. An exhaustive list and detailed description of the categories are provided in Additional file 2. Additionally, we created 36 subcategories of foods (22 for general foods, 7 for fats, oils, nuts and seeds, and 7 for beverages) to explore Nutri-Scores’ ability to discriminate within categories. This categorization was based on the standard food categories in the food database and were considered relevant for aiding consumers toward healthier foods (e.g., breads, breakfast cereals) and to assess Nutri-Scores’ ability to classify foods in accordance with the Norwegian FBDGs (e.g., red meat vs. poultry). The miscellaneous food category consisted of general foods that were too few to gather in a separate category or hard to place in other categories. All subcategories were mutually exclusive, and details are reported in Additional file 3.

### Statistical analyses

#### Nutri-Scores’ discriminatory ability of nutritional quality of foods within categories

This study used descriptive statistics and boxplots to explore the updated Nutri-Scores’ ability to discriminate the nutritional quality of foods within food categories. Boxplots and dots indicating individual foods were used to display the distribution of Nutri-Score within categories. We present the distribution (n and percent) of Nutri-Score classes within each food category in additional files. The discriminatory ability was pragmatically assessed by considering the most frequent Nutri-Score class and the number of available classes within each food category, for both main- and subcategories of foods. Having products in three or more classes within a category was considered acceptable to be able to discriminate between foods, similar to previous studies [17, 25–27]. In the main text we primarily present results for subcategories, while the results for main categories of foods are placed in additional files. Nutri-Score for all single foods within each food category were explored to look for irregularities. Descriptive statistics (median, interquartile range (IQR), minimum and maximum) were calculated to determine the points allocated by *each* component in the Nutri-Score algorithms. These statistics were calculated overall for each algorithm and for categories of foods to investigate the relative contribution of each component to the total points within each food category.

#### Assessment of Nutri-Scores’ ability to classify foods in accordance with the Norwegian food-based dietary guidelines

The Norwegian FBDGs were developed from a review of systematic reviews and evaluation of the quality of evidence of the association of foods and nutrients with obesity and chronic diet-related diseases, with the aim to prevent these diseases in the Norwegian population [28]. A brief description of the development of the FBDGs is presented in Additional file 4. We pragmatically assessed the updated Nutri-Scores’ ability to classify foods in accordance with the Norwegian FBDGs by examining the Nutri-Score for subcategories of foods specifically mentioned in the guidelines [29] by using the aforementioned descriptive statistics. We expected lower total points or predominantly Nutri-Score class A or B for recommended foods like fruit, berries, vegetables, and fish, and higher total points or mainly Nutri-Score class D or E for foods we should limit e.g., red and processed meat, and foods high in salt and sugar, like processed foods, sugar-sweetened beverages, and candy. Only subcategories of foods mentioned in the Norwegian FBDGs were explored to assess Nutri-Scores’ accordance with the guidelines. E.g., plant-based alternatives to dairy products, which are not mentioned in the FBDGs, were not included in this assessment.

To structure the assessment, we created a table with the Norwegian FBDGs where we commented upon Nutri-Scores’ classification of foods in accordance with each FBDG based on all results for each relevant food category or specific foods. The FBDGs recommend limiting intake of red and processed meat but also specify to choose lean meat and lean meat products [29]. We interpret this as although red meat should be limited, if choosing red meat, one should choose lean red meat instead of red meat with a higher fat content or processed red meat. Authors ultimately did a pragmatic, overall assessment to conclude on Nutri-Scores’ ability to classify foods in accordance with the Norwegian FBDGs.

We also investigated agreement between Nutri-Score and the Norwegian Bread Scale label for breads, using information available in the food database. The Bread Scale label is a voluntary label for breads helping consumers to choose breads with more whole grains, by indicating the coarseness based on the percentage of whole grains, wholewheat meal flour and bran from the total amount of flour in the bread [30]. The label has four coarseness categories: 0–25.9% (white bread), 26–50.9%, 51–75.9%, and 76–100% (extra coarse bread). We tested the agreement using a 2-sided chi-square test with a significance level of 5%. Statistics analyses were conducted using Stata (version 17.0) and figures were made in R version 4.2.2.

#### Ethics

Ethical approval was not required as no human or animal subjects were involved in this study.

## Results

### Discrimination of nutritional quality of foods using the updated Nutri-Score

Of the 1,782 foods and beverages in the sample, 27% were classified with Nutri-Score A, 12% with B, 23% with C, 21% with D, and 17% with E (Additional files 5﻿ and ﻿6).

**Fig. 1:**
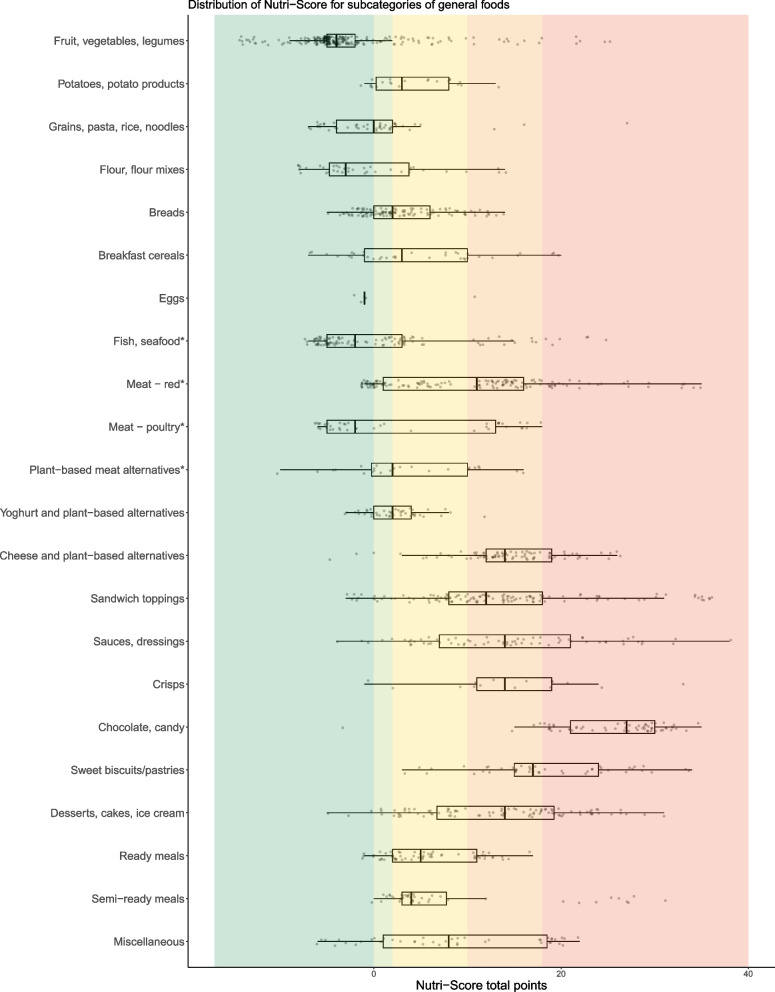
Distribution of Nutri-Score for subcategories of general foods (*n* = 1468). Distributions are shown with boxplots where the vertical line in the box represent the median total points, the box indicate the 25^th^ and 75^th^ percentile and the whiskers indicate the lowest or highest value (maximum higher or lower than 1.5 × the interquartile range). The dots represent all single products within the category. Dark green color background indicates Nutri-Score class A, light green Nutri-Score class B, yellow Nutri-Score class C, light orange Nutri-Score class D, and dark orange indicates products classified with Nutri-Score E. *Excluding typical spreads or cold cuts used as sandwich toppings as they are included in the sandwich toppings category

**Fig. 2:**
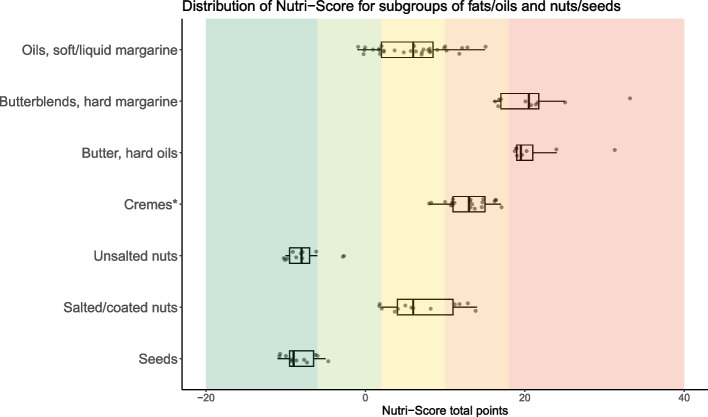
Distribution of Nutri-Score for subcategories of fats/oils and nuts/seeds (*n* = 105). Distributions are shown with boxplots where the vertical line in the box represent the median total points, the box indicate the 25^th^ and 75^th^ percentile and the whiskers indicate the lowest or highest value (maximum higher or lower than 1.5 × the interquartile range). The dots represent all single products within the category. Dark green color background indicates Nutri-Score class A, light green Nutri-Score class B, yellow Nutri-Score class C, light orange Nutri-Score class D, and dark orange indicates products classified with Nutri-Score E. *Creams: used for cooking, such as regular cream, crème fraiche, sour cream, and plant-based cream alternatives

**Fig. 3:**
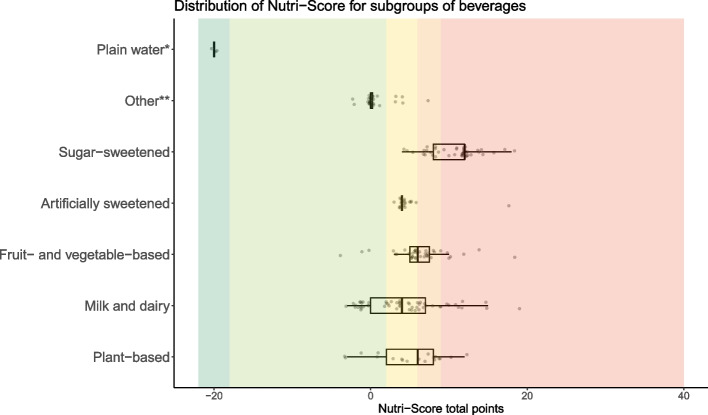
Distribution of Nutri-Score for subcategories of beverages (*n* = 209). Distributions are shown with boxplots where the vertical line in the box represent the median total points, the box indicate the 25^th^ and 75^th^ percentile and the whiskers indicate the lowest or highest value (maximum higher or lower than 1.5 × the interquartile range). The dots represent all single products within the category. Dark green color background indicates Nutri-Score class A (water), light green Nutri-Score class B, yellow Nutri-Score class C, light orange Nutri-Score class D, and dark orange indicates products classified with Nutri-Score E. *Plain water is not given total points but is included for illustrative purposes. **Other beverages include carbonated and flavored water, tea, coffee, and non-alcoholic wine/beer

The distribution of Nutri-Score by main categories is presented in Additional files 5 and ﻿7, while results for the subcategories are presented below and in Additional file 6. Figures 1, 2 and 3 illustrate differences in Nutri-Score both within and between the subcategories of foods.

There were foods in at least three out of the five Nutri-Score classes in all subcategories of general foods, except for *eggs* with foods in only two Nutri-Score classes. The majority of *fruits, vegetables and legumes* (85%); *grains, pasta, rice and noodles* (84%); *flour and flour mixes* (71%); and *eggs* (80%) were classified with Nutri-Score A or B (Additional file 6). Most *cheeses and plant-based alternatives* to cheese (85%); *crisps* (81%); *chocolate/candy* (98%); and *sweet biscuits/pastries* (89%) were classified with Nutri-Score D or E.

*Oils and soft/liquid margarines* and *salted/coated nuts* had foods in more than three of the Nutri-Score classes. All *butter, butterblends and hard margarine and oils* were classified with either Nutri-Score D or E, while most (87%) *oils and soft/liquid margarine* were classified with B or C (Additional file 6). *Cremes* were classified with Nutri-Score C or D. *Seeds and unsalted nuts* were mostly classified with Nutri-Score A (91% and 82% respectively).

For all subcategories of beverages, there were products in at least two of the four possible classes of Nutri-Score. More than 80% of products in the *other beverages* category were classified with Nutri-Score B, 95% of *artificially sweetened beverages* were classified with Nutri-Score C and most (93%) of *sugar-sweetened beverages* were classified with D or E (Additional file 6), whereas there was a larger distribution of Nutri-Score classes for the remaining subcategories of beverages.

Figures 1, 2 and 3 reveal evident outliers within subcategories. In the *fruit, vegetables, and legume* category, which is generally considered as healthy, products like fruit or vegetables in oils, dried or in powder forms with a higher content of energy and/or sugar and/or salt were classified with Nutri-Score E. In the *crisps* and *chocolate/candy* categories, which is generally considered as unhealthy, products like popcorn without added fat and salt containing a high content of protein and fiber; and sugar free drops containing a lower energy and a higher fiber content were classified with Nutri-Score A. A detailed description of Nutri-Score for foods in the specific subcategories including outliers are in Additional file ﻿8.

### Contribution of points from each component in the Nutri-Score algorithms

The overall central and dispersion of points for each component in the three Nutri-Score algorithms are shown in Table 2**,** and for categories in Additional file ﻿9. In our dataset, salt (median points: 2 (IQR: 6)), energy (2 (3)), saturated fat (1 (4)) and protein (2 (3)) were the main determinants of variation in the Nutri-Score total points for general foods. Salt provided the largest variation in points for many food categories, particularly for *cheese, meat, fish, sandwich toppings, sauces and dressings, crisps, and miscellaneous* products (Additional file 9). Regarding proportion of fruit, vegetables and legumes, this component primarily allocated points within the *fruit, vegetables and legumes category*.

**Table 2:** Central and dispersion of points allocated by each component in the Nutri-Score algorithms

	Nutri-Score algorithm for general foods (*n* = 1468)		Nutri-Score algorithm for fats, oils, nuts and seeds (*n* = 105)		Nutri-Score algorithm for beverages (*n* = 206)^b^	
	Median (IQR)	min – max	Median (IQR)	min – max	Median (IQR)	min – max
**Points from unfavorable components**						
Energy (0–10 points)	2 (3)	0—9	NA	NA	3 (3)	0—10
Energy from saturated fat (0–10 points)	NA	NA	2 (4)	0—10	NA	NA
Sugars (0–15 points)	0 (2)	0—15	0 (1)	0—6	4 (7)	0—10
Saturated fat (0–10 points)	1 (4)	0—10	NA	NA	0 (0)	0—5
Saturated fat/total fat (0–10 points)	NA	NA	2 (8)	0—10	NA	NA
Salt (0–20 points)	2 (6)	0—20	0 (4)	0—14	0 (0)	0—2
NNS (absence = 0 points / presence = 4 points)	NA	NA	NA	NA	0 (0)	0—4
**Points from favorable components**						
Protein (0–7 points)	2 (3)	0—7	0 (6)	0—7	0 (6)	0—7
Fiber (0–5 points)	0 (1)	0—5	0 (5)	0—5	0 (0)	0—5
FVL-proportion (0,1,2 or 5/6^a^ points)	0 (0)	0—5	0 (0)	0—5	0 (0)	0—6

^a^6 points for beverages

^b^Plain water was excluded as it is not calculated but automatically classified with Nutri-Score A

FVL: Fruit, vegetables and legumes; IQR: interquartile range; NA: Not applicable; NNS: non-nutritive sweeteners

The main components providing variation in the total points for fats, oils, nuts and seeds were energy from saturated fat (2 (4)), saturated fat/total fat proportion (2 (8)), salt (0 (4)), protein (0 (6)) and fiber (0 (5)). For beverages, sugar (4 (7)) and energy (3 (3)) components contributed to the most points and variation in total points, while protein provided variation in favorable points (IQR: 6). More details in Additional file 9.

### Nutri-Scores’ ability to classify foods in accordance with the Norwegian food-based dietary guidelines

The Norwegian FBDGs promote increased intake of fruit, berries, vegetables, and fish [29]. Approximately 85% of the foods in the *fruit, vegetables, and legumes* category were classified with Nutri-Score A or B (Additional file 6). In the *fish* category, 65% of the foods were classified with A or B. Moreover, the FBDGs specify to choose *water* as a thirst-quencher, which was the only beverage classified with Nutri-Score A in the updated Nutri-Score.

The FBDGs recommend limiting intake of red and processed meat and foods high in sugar and salt [29]. Over half of foods in the *red meat* category were classified with Nutri-Score D or E (Additional file 6) and most of these meats had a higher fat and/or salt content. Processed meats like sausages, bacon, cured meat, and meat patties, from both red meat and poultry, were more frequently classified with Nutri-Score D and E than unprocessed meats (Additional file 8). More than 80% of salty or sugary foods like *crisps* and *chocolate/candy*, were classified with Nutri-Score D or E (Additional file 6). Moreover, 93% of *sugar-sweetened beverages* were classified with Nutri-Score D or E.

**Fig. 4:**
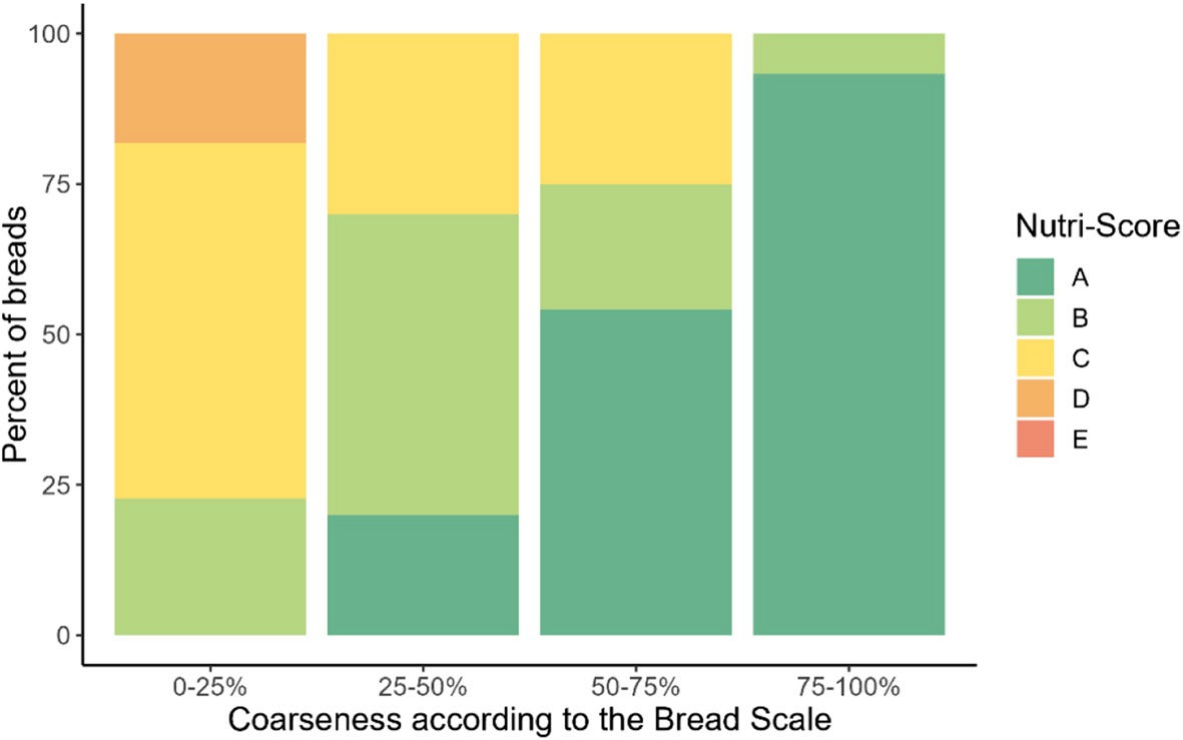
Breads (*n* = 71) categorized according to the Bread Scale label and the distribution of the updated Nutri-Score. The Bread Scale label indicates coarseness by the proportion of the flour in breads that is wholegrain, wholegrain flour and bran [30]

Within certain food groups like cheeses, cooking cremes, processed meats (sausages), ready meals and pasta/rice, the updated Nutri-Score did not capture differences consistent with the Norwegian FBDGs. For example, regular semi-hard cheese and cremes and their low-fat equivalent, sausages and their leaner versions, and ready meals with red meat versus white meat/fish, in many cases get the same Nutri-Score class (details in Table 3 and Additional file 8). Moreover, the updated Nutri-Score did not discriminate between whole grain and refined rice/pasta as all were classified with Nutri-Score A, also observed for flours and flour mixes (Additional file 8). A significant (*p* < 0.001) association was found between Nutri-Score and the Bread Scale. More than 90% of breads with 75–100% coarseness were classified with Nutri-Score A, while 77% of breads with 0–25% coarseness were classified with Nutri-Score C or D (Fig. 4 and Additional file ﻿10). Additional details of how the updated Nutri-Score aligned with each Norwegian FBDG is presented in Table 3.

**Table 3:** Assessment of the updated Nutri-Score and its accordance with the Norwegian food-based dietary guidelines (FBDGs) [29, 31, 32]

Norwegian FBDGs, including relevant specifications	The updated Nutri-Score and the Norwegian FBDGs
**Eat at least five portions of vegetables, fruit and berries every day.** Not including potatoes and legumes and nuts. Vary between different fruit and vegetables. Choose boiled and baked potatoes over fried. Eat a handful of unsalted nuts every day. One glass of juice can be one of the daily fruit and vegetable portions.	Over 80% of products in the fruit, vegetables and legumes category were classified with Nutri-Score A or B (Additional file 6). Comparing the distribution of Nutri-Score for the fruit, vegetables and legumes category to the other food categories, most products were clustered in Nutri-Score class A (Fig. 1 and Additional file 7). The fruit, vegetables and legumes component mainly allocated points within the fruit, vegetables and legumes category (Additional file 9). In general, potato fries and potato products were classified with Nutri-Score C or D, whereas raw or boiled potatoes were classified with Nutri-Score A or B (Additional file 8). Unsalted nuts were mainly classified with Nutri-Score A (82%) and most salted/coated nuts were classified with Nutri-Score C (46%) (Fig. 2 and Additional file 6). Approximately half of juices (48%) were classified with Nutri-Score C (Additional files 6 and 8).
**Eat whole grain foods every day.** Whole grain cereals/grains should provide 70–90 g wholegrain wheat or whole grain every day. Choose grain products with a high content of fiber and whole grain, and low content of fat, sugar and salt. Examples of food that can contribute with whole grain are breads, breakfast cereals, oat porridge, whole grain pasta or rice.	Cereals, grains and products thereof were distributed across all Nutri-Score classes (Additional files 5 and 7). A total of 51% of grains, pasta, rice and noodles were classified with Nutri-Score A (Additional file 6), but the Nutri-Score classes did not differentiate between whole grain and refined pasta or rice as both got Nutri-Score A (Additional file 8). Close to 60% of breads were classified with Nutri-Score A or B (Additional file 6). Fiber was the component allocating the most favorable points, and variation in points, in the updated Nutri-Score for flour, flour mixes, breads and breakfast cereals (Additional file 9). Salt contributed with unfavorable points in breads, while sugars was influential among breakfast cereals. There was a significant association between Nutri-Score and the Bread Scale, and more than 90% of the breads with 75–100% coarseness were classified with Nutri-Score A (Fig. 4 and Additional file 10). Half of breakfast cereals were classified with Nutri-Score A or B (Additional file 6). Two out of 20 breakfast cereals with more than 10 g of sugars per 100 g were classified with Nutri-Score A (Additional file 8). Sugary grain products, such as sweet biscuits/pastries, were mostly (85%) classified with D or E (Additional file 6), and sugars and saturated fat were the most influential components (Additional file 9).
**Eat fish two to three times a week. You can also use fish as a sandwich topping.** Approximately 50% should be fatty fish (such as salmon, trout, mackerel, herring, eel, halibut, sardine).	Most (54%) fish products were classified with Nutri-Score A (Additional file 6). Both lean and fatty fish could be classified with Nutri-Score A (Additional file 8).
**Choose lean meat and lean meat products. Limit the amount of processed meat and red meat.** Limit the amount of red meat and processed red meat to less than 500 g per week. Choose poultry, lean meat and lean meat products that are low in salt. Limit the amount of processed meat that are smoked, salted or preserved using nitrate or nitrite, such as bacon or cured sausage.	Overall, red meat products were classified with less favorable Nutri-Scores than poultry (Fig. 1), and 53% of red meat and 38% of poultry products were classified with Nutri-Score D or E (Additional file 6). However, lean red meat products could achieve Nutri-Score classes A and B. For red meat and poultry, saturated fat and salt were the components allocating the most unfavorable points, and variation in points, in the updated Nutri-Score (Additional file 9). Red meat products classified with Nutri-Score A or B, were mainly raw, unprocessed meats (Additional file 8). In general, unprocessed and lean meats were more frequently classified in better Nutri-Score classes than processed and meats with higher fat content. Unprocessed meats from both red meat and poultry were generally classified with better Nutri-Scores than processed meats. Salted and preserved meats from both red meat and poultry, such as bacon and sausages were mainly classified with Nutri-Score D or E.
**Include low-fat dairy foods in your daily diet.** Milk and cheese are commonly consumed, so choose the leaner options for everyday use. Limit the use of dairy products containing high levels of saturated fat, such as whole milk, full-fat cream, full-fat cheese, and butter. Choose dairy products that are low in fat, salt and added sugar. Choose low-fat milks, such as skimmed or partly skimmed milk (≤ 0.7% fat).	Dairy products (excluding creams and milk) were distributed across all Nutri-Score classes, however most (38%) were classified with Nutri-Score D (Additional files 5 and 7). Approximately 86% of cremes were classified with Nutri-Score D, whereas milk and dairy-based beverages were mainly classified with Nutri-Score B (38%) or C (35%) (Additional file 6). Overall, the updated Nutri-Score does not seem to capture the difference in fat content within certain categories of dairy foods, such as cremes and cheeses (Additional file 8). A total of 38% of milk and dairy-based beverages were classified with Nutri-Score B (Additional file 6). Products classified with Nutri-Score B were generally low-fat, such as skimmed (0.1% fat) or partly skimmed milk (0.5–1.2% fat) and with no or low added sugar content (Additional file 8). Approximately 30% of yoghurts were classified with Nutri-Score A (Additional file 6). Sugars and saturated fat were the main unfavorable components allocating points within this category (Additional file 9). Most cheeses and their plant-based alternatives (58%) were classified with Nutri-Score D (Additional file 6). The updated Nutri-Score did not consistently capture large differences in saturated fat content between cheeses as most cheeses with varying saturated fat content were classified with Nutri-Score D, also indicated by the lack of variation in points from saturated fat in the algorithm, i.e. saturated fat contributed with no variation in unfavorable points among cheeses (Additional file 9). Approximately 86% and 14% of cremes were classified with Nutri-Score D and C respectively (Additional file 6), and no products got a Nutri-Score A or B. In general, full-fat cremes and their low-fat options were classified with D (Additional file 8).
**Choose cooking oils, liquid margarine and soft margarine spreads instead of hard margarines and butter.** Replace foods high in saturated fats with foods containing more unsaturated fats.	Fats and oils were mostly categorized with Nutri-Score D (37%) and 27% with Nutri-Score C (Additional file 5). No products in this category got Nutri-Score A. For subcategories of fats/oils, 100% of butter, butterblends and hard margarine and oils were classified with either Nutri-Score D or E, whereas most (87%) oils and soft/liquid margarine were classified with Nutri-Score B or C (Additional file 6).
**Choose foods that are low in salt and limit the use of salt when preparing food and eating.** Choose foods and ready meals with less salt.	Overall, salt was the component contributing with the largest variation in points for many food categories and was the component providing the most unfavorable points (Table 2 and Additional file 9). Over 80% of crisps were classified with Nutri-Score D or E (Fig. 1 and Additional file 6). For ready meals, salt was the component providing the most points and the saturated fat was component providing most variation in points (Additional file 9).
**Avoid foods and drinks that are high in sugar.** Limit consumption of squash, soda, nectar, sweet cookies, sweet pastries, chocolate, and candy.	Approximately 90% of chocolate/candy were classified with Nutri-Score E (Additional file 6). Sweet biscuits/pastries were mostly (89%) classified with D or E (Additional file 6). Respectively, 66% and 27% of sugar-sweetened beverages were classified with Nutri-Score E and D (Additional file 6).
**Choose water as a thirst-quencher.** Choose water when thirsty. Limit sugar-sweetened beverages in everyday life. Other beverages: Choose low-fat/lean milk (≤ 0.7% fat); avoid a high intake of juice, one glass of juice can be counted as one of the daily recommended portions of fruit and vegetables.	Plain water was the only beverage classified with Nutri-Score A. Nutri-Scores for other beverages are described above.

FBDGs: Food-based dietary guidelines

## Discussion

When applied in a Norwegian context, the updated Nutri-Score had an overall acceptable discriminatory ability of the nutritional quality of foods within food categories. In almost all food categories there were products in at least three of the five Nutri-Score classes. The foods content of salt and the beverages’ content of sugar were the most influential components to the updated Nutri-Scores.

Overall, the authors assessed that the updated Nutri-Score had the ability to classify foods in accordance with the Norwegian FBDGs in most cases because 1) foods the Norwegian FBDGs recommend to consume more of (i.e. fruit, berries, vegetables, whole grain products and fish) [29], were in general classified with Nutri-Score A or B; 2) the updated algorithms included nutrients or components that the FBDGs directly or indirectly specify to increase or limit the intake of (such as fruit and vegetables, sugar, salt, and indirectly saturated fat through dairy products and red meat); and 3) foods the Norwegian FBDGs recommend to decrease or limit intake of were mainly classified with Nutri-Score D or E (such as processed meat, red meat with a higher saturated fat and/or salt content, crisps, chocolate/candy, and sugar-sweetened beverages). However, we could not conclude that Nutri-Score had the ability to perfectly classify foods in accordance with the Norwegian FBDGs as we observed some inconsistencies such as Nutri-Scores’ inability to differentiate between full-fat cheeses and cremes or processed meats (sausages) and their low-fat or leaner equivalent, and between whole grain and refined pasta/rice.

### Nutri-Score and its discriminating ability of nutritional quality

The updated Nutri-Score has so far been scarcely applied in the scientific literature [33, 34], and to our knowledge no study has evaluated the three updated Nutri-Score algorithms.

Nutri-Score should help consumers in choosing healthier foods by discriminating between foods within categories [14]. We observed that most subcategories of food and beverages had products in at least three Nutri-Score classes indicating acceptable discriminatory ability, except for very specific categories, such as eggs, seeds, unsalted nuts, butters, hard margarines and oils, and artificially sweetened beverages, which had products in one or two of the five Nutri-Score classes. This was likely due to product homogeneity. Julia et al. considered two classes as satisfactory discrimination for similar products from different brands [27]. However, several studies, including the current study, used the availability of products in three Nutri-Score classes (out of five) within each food category as an acceptable measure of discriminatory performance [17, 25–27]. In the current study, Nutri-Scores’ ability to discriminate between products can depend on the definition of food categories and the similarity of the products within these categories. When assessing the discriminative ability of Nutri-Score for fats/oils, it may be more reasonable to differentiate between products in the overall category (presented in Additional file 7) rather than its subcategories. It is important to consider the various ways of categorizing foods when evaluating Nutri-Scores’ discriminatory ability. Larger categories tend to exhibit better discriminatory capacity, while very homogeneous categories like eggs may have limited discriminative capacity, as noted by previous studies [26, 35]. Notably, certain food categories may have minimal discriminatory performance, with foods clustering in one or two Nutri-Score classes, which can be acceptable. This is particularly relevant for nutritious foods like plain fruits and vegetables, as well as foods containing nutrients that should be limited, such as chocolate and candy.

Nutri-Score’s ability to discriminate between nutritional quality of foods is linked to the algorithm’s components. In our study, we explored which components that contributed to variation in the Nutri-Score. We found that salt allocated many points, and variation in points, for several food categories (Additional file 9), indicating salts’ influence in the calculation of the updated Nutri-Score. This finding was not surprising as salt was the component that could provide the most unfavorable points in the updated algorithms [20, 21]. The Scientific Committee of the Nutri-Score report that salt has been adjusted in the updated algorithm to better differentiate highly salted foods and promote reformulation [19]. The European Food Safety Authority acknowledge the use of components to adequately differentiate the nutritional quality of foods in nutrient profiles [36].

### The updated Nutri-Scores’ ability to classify foods in accordance with the Norwegian food-based dietary guidelines

Some of the components used to calculate the updated Nutri-Score are specified in the Norwegian nutritional recommendations and the FBDGs. We found that, the updated Nutri-Score in most cases classified foods in accordance with the Norwegian FBDGs. Pitt et al. assessed agreements and disagreements between the Nordic Keyhole, which is a label in line with the Norwegian FBDGs supported by the Norwegian Directorate of Health, and the updated Nutri-Score for foods on the Swedish market [33]. Pitt et al. concluded that in general there is a good level of agreement between these labels. This somewhat reflect our findings, but as their aim and food categories were different from ours, there are limitations in comparing these studies. We observed some specific inconsistencies, discussed below.

Grains, pasta, rice, and noodles are potential sources of whole grain and fiber, recommended to consume daily according to the Norwegian FBDGs [29]. In our study, the majority (84%) of these foods received Nutri-Score A or B. However, the updated Nutri-Score did not consistently differentiate between refined and whole grain pasta/rice, as well as flours, as all were classified with Nutri-Score A. These findings are in line with Pitt et al. reporting disagreements between the Keyhole label and the updated Nutri-Score for cereal and grain-based products [33]. Also, the Scientific Committee of Nutri-Score acknowledged this limitation but prioritized discriminating between whole and refined grain bread over pasta and rice for fiber intake in European countries [20]. While Nutri-Score aligns well with the Norwegian FBDGs for breads, discriminating between other whole and refined grain products are also important as the recommendations specify to choose whole grain over refined grain products [29].

For fish, which is recommended in the FDBGs [29], we found that 65% of fish products were classified with Nutri-Score A or B. Some fish products received Nutri-Score D or E due to their relatively high salt and energy content, particularly fatty fish. Pitt et al. reported that 12% of the fish products with Nutri-Score C, D, or E were eligible for the Keyhole label [33]. The latter, and the recommendation from the Norwegian Directorate of Health to choose Keyhole products, suggest that, for fish, the focus should be on consuming fish itself rather than solely considering the content of other unfavorable components like salt.

The updated Nutri-Score seems to capture the recommendation to choose less red meat as red meats scored more poorly than poultry and fish [29]. Additionally, unprocessed and less processed meats were generally classified with better Nutri-Score classes than highly processed meats (Additional file 8). Yet, red meat could obtain a Nutri-Score A which 22% of products in the *red meat* category did, which one might not expect for products the FBDGs recommend to limit. However, red meat classified with Nutri-Score A were unprocessed and lean which can be in line with the Norwegian FBDGs. Still, one may question why this proportion was relatively high and if red meat should be able to achieve Nutri-Score class A. If our dataset contained a larger proportion of processed meat or red meat with a higher fat content, this proportion would likely be lower and the proportion of red meat classified with Nutri-Score D or E would be higher.

Processed meat from poultry should ideally have a better Nutri-Score than processed red meat, which is not necessarily the case due do other components affecting the Nutri-Score total points. For example, we observed that poultry sausages containing more salt and red meat sausages were both classified with Nutri-Score class D. The updated algorithm seems to capture the differences between these products in the total points, and the lack of differentiation may be attributed to the Nutri-Score class thresholds and the limited number of classes. According to the Norwegian FBDGs [29], sausages, independent of the meat source, are processed meat that should be limited in general. Therefore, processed meat should be classified with Nutri-Score D or E to align with this recommendation. A limited intake of 500 g of red meat and processed red meat per week is also specified in the FBDGs [29], indicating a difference between processed meats depending on the source. To align with the specific Norwegian FBDGs and aid consumers, there should arguably be more variation in Nutri-Score classes among sausages. This concept extends to other food categories, like ready meals. Ready meals without red meat or with poultry should possibly receive a better Nutri-Score than similar ready meals containing red meat. The updated Nutri-Score might not adequately differentiate between ready meals with and without red meat (Additional file 8), thereby deviating somewhat from the recommendation to limit red meat consumption.

We observed inconsistencies between certain dairy products and the recommendation to choose low-fat over full-fat products [29]. In our analysis of the updated Nutri-Score algorithms, we observed that saturated fat influenced the total points for most foods but not for cheeses, likely due to their higher saturated fat content. In our study, 78% of cheeses exceeded the threshold of 10 g of saturated fat per 100 g, which is not differentiated by the Nutri-Score. For this reason, both low-fat (16% fat) and full-fat (26% fat) versions of commonly consumed semi-hard cheeses received the identical Nutri-Score class (Nutri-Score D). Pitt et al. 2023 also mentioned this example, as low-fat cheese was eligible for the Keyhole and classified with Nutri-Score D [33]. We observed similar inconsistencies for cooking cremes. Altogether, these findings suggest that the updated Nutri-Score might not discriminate the difference in nutritional quality based on fat content for cheeses and cremes. Because cheeses are a big source of saturated fat in the Norwegian diet [37], it is important for the updated Nutri-Score to capture these differences to align with the Norwegian FBDGs. On the other hand, a few low-fat cheeses were classified with Nutri-Score A, indicating their higher nutritional quality and providing options for consumers. This example illustrates the complexity of setting thresholds across food categories, as category-specific thresholds would likely better discriminate nutritional quality [38, 39].

The updated Nutri-Score seems to capture the recommendation to choose cooking oils and soft margarine over butter, as the latter was classified with Nutri-Score E and oils and soft margarines were with B and C. Also, as previously discussed, Nutri-Score seemed to capture the difference in salt content for foods, discouraging products with a high salt content. Furthermore, Nutri-Scores’ classification of crisps, chocolate/candy, and sweet biscuits/pastries as D or E is in line with the Norwegian FBDGs as these are recommended to limit the consumption of [29].

### Implications and generalizability

An across-the-board algorithm such as Nutri-Score [4] might not perfectly discriminate between products within food-specific categories, and some small level of inconsistencies might be expected. The updated Nutri-Score will unlikely contradict the Norwegian FBDGs if implemented in Norwegian stores, yet some confusion or lack of aiding consumers choosing healthier food alternatives within a food category might occur. It is important to highlight that Nutri-Score is a tool to aid consumers in choosing single foods and the FBDGs are guidelines toward healthier diets [22]. The approaches to affect population diets are complementary and Nutri-Score should be accompanied with the FBDGs.

Our study is from a Norwegian context, thus generalization of our findings to countries with different FBDGs, and countries with other foods and using vast different foods categories, should be conducted with caution. Studies applying the updated Nutri-Score in other countries are needed to assess the updated Nutri-Score in various contexts and to ensure it classifies foods in accordance with different FBDGs, particularly if the updated Nutri-Score is implemented as the harmonized FoPNL in the European Union.

### Strengths and limitations

The main limitation in the current study is the use of a food database containing mostly generic foods, lacking the markets share of products and many brand-specific foods currently sold in Norwegian stores. For example, there were 16 products in the *salty snacks* category (crisps, popcorn, tortilla chips, etc.) representing common products, including several brand-specific products. However, this number of products does not reflect all salty snack products found in Norwegian stores. Our salty snacks data contain approximately one product from each producer, but in stores each producer offer several similar products with small differences in nutritional content (e.g., salt and protein). Hence, we might not show the real distribution of Nutri-Score within food categories which further could limit capturing inconsistencies with the updated Nutri-Score and its accordance with the Norwegian FBDGs. Nevertheless, the database contains a representative number of foods likely sufficient to evaluate Nutri-Scores’ discriminatory ability of the nutritional quality of foods in a Norwegian setting, as well as its ability to classify foods in accordance with the Norwegian FBDGs, as this database is used in national dietary surveys representative of the Norwegian population. Furthermore, the currently used food database provides information on fiber and many recipes for foods which are needed when calculating Nutri-Score, thus limiting errors and misclassification.

An inherent problem when evaluating nutrient profiling models, such as Nutri-Score, is the lack of a gold standard for defining a healthy food [9] and no consensus on the best way to evaluate alignment between nutrient profiles or FoPNLs and FBDGs. For this reason, we used a pragmatic approach by examining the Nutri-Score for subcategories of foods specifically mentioned in the guidelines, which also has limitations as not all foods within a food category are specifically mentioned in the Norwegian FBDGs. Even though we tried to be transparent in our assessment, this approach could have resulted in discretionary assessments. Also, additional inconsistencies may not be captured using the methods in this study. We are currently conducting a qualitative study of Nutri-Score among stakeholders in the Norwegian food system to capture additional strengths and limitations of the updated Nutri-Score in a Norwegian context.

## Conclusion

This study is the first to evaluate the complete updated Nutri-Score. We observed an overall acceptable discriminatory performance of the Nutri-Score in a Norwegian setting. In most cases the updated Nutri-Score was in accordance with the Norwegian FBDGs, however, we observed inconsistencies related to inabilities to discriminate between whole versus refined grain products and between cheeses, cremes and processed red meats (sausages) with different fat content. Our evaluation suggests that together with the FBDGs, the updated Nutri-Score could be a useful tool in guiding consumers toward healthier food choices in a Norwegian setting, but the use and effect of the updated Nutri-Score among Norwegian consumers remains to be evaluated.

### Supplementary Information

**Additional file 1.** The updated Nutri-Score algorithms.

**Additional file 2.** Main food categories.

**Additional file 3.** Subcategories of foods.

**Additional file 4.** The development of the Norwegian food-based dietary guidelines.

**Additional file 5.** Distribution of Nutri-Score classes for main food categories.

**Additional file 6.** Distribution of Nutri-Score classes for subcategories of general foods.

**Additional file 7.** Distribution of Nutri-Score for main categories of foods.

**Additional file 8.** Detailed description of the updated Nutri-Score for subcategories of foods.

**Additional file 9.** Central and dispersion of points from each component in the Nutri-Score algorithms within food categories.

**Additional file 10.** Breads with the bread scale and Nutri-Score.

## Data Availability

The dataset generated and/or analyzed during the current study are not publicly available due to the data being proprietary (University of Oslo) but a subset of the dataset is available from the corresponding author on reasonable request.

## Abbreviations

FBDGs: Food-based dietary guidelines
FoPNLs: Front-of-pack nutrition labels
IQR: Interquartile range
WHO: World Health Organization
