# Supplementary material for: How does the updated Nutri-Score discriminate and classify the nutritional quality of foods in a Norwegian setting?
Source: Int J Behav Nutr Phys Act. 2023 Oct 10;20:122. doi: 10.1186/s12966-023-01525-y (PMC10563306; doi:10.1186/s12966-023-01525-y)
Supplement: Supplementary file 1 — Additional file 1. The updated Nutri-Score algorithms. [file 12966_2023_1525_MOESM1_ESM.docx]

**Additional file 1. The updated Nutri-Score algorithms**

| **Nutri-Score algorithm for general foods^[[1]](#footnote-2)^** | | | | | | | |
| --- | --- | --- | --- | --- | --- | --- | --- |
| Points | Unfavorable components | | | | Favorable components | | |
|  | Energy  (kJ per 100g) | Sugars (g per 100g) | Saturated fat (g per 100g) | Salt  (g per 100g) | Protein*  (g per 100g) | Fiber  (g per 100g) | Fruit, vegetables, legumes (%) |
| 0 | ≤ 335 | ≤ 3.4 | ≤ 1.0 | ≤ 0.2 | ≤ 2.4 | ≤ 3.0 | ≤ 40 |
| 1 | > 335 | > 3.4 | > 1.0 | > 0.2 | > 2.4 | > 3.0 | > 40 |
| 2 | > 670 | > 6.8 | > 2.0 | > 0.4 | > 4.8 | > 4.1 | > 60 |
| 3 | > 1005 | > 10 | > 3.0 | > 0.6 | > 7.2 | > 5.2 | - |
| 4 | > 1340 | > 14 | > 4.0 | > 0.8 | > 9.6 | > 6.3 | - |
| 5 | > 1675 | > 17 | > 5.0 | > 1.0 | > 12 | > 7.4 | > 80 |
| 6 | > 2010 | > 20 | > 6.0 | > 1.2 | > 14 |  |  |
| 7 | > 2345 | > 24 | > 7.0 | > 1.4 | > 17 |  |  |
| 8 | > 2680 | > 27 | > 8.0 | > 1.6 |  |  |  |
| 9 | > 3015 | > 31 | > 9.0 | > 1.8 |  |  |  |
| 10 | > 3350 | > 34 | > 10 | > 2.0 |  |  |  |
| 11 |  | > 37 |  | > 2.2 |  |  |  |
| 12 |  | > 41 |  | > 2.4 |  |  |  |
| 13 |  | > 44 |  | > 2.6 |  |  |  |
| 14 |  | > 48 |  | > 2.8 |  |  |  |
| 15 |  | > 51 |  | > 3.0 |  |  |  |
| 16 |  |  |  | > 3.2 |  |  |  |
| 17 |  |  |  | > 3.4 |  |  |  |
| 18 |  |  |  | > 3.6 |  |  |  |
| 19 |  |  |  | > 3.8 |  |  |  |
| 20 |  |  |  | > 4.0 |  |  |  |
| *Red meat products are given maximum 2 protein points. | | | | | | | |

**Nutri-Score for general foods calculation:**

Summarize points from unfavorable components.

If unfavorable component is ≥ 11 points, then apply formula:
Nutri-Score total points = total unfavorable components points - (points from the fiber component + points from the fruit, vegetables and legumes component)

If unfavorable component is < 11 points or the food is, then apply formula:
Nutri-Score total points = total unfavorable component points - total favorable components points

Classify Nutri-Score using the table below.

| Nutri-Score total points for general foods | Class | Color |
| --- | --- | --- |
| ≤ 0 | A | Dark green |
| 1 to 2 | B | Light green |
| 3 to 10 | C | Yellow |
| 11 to 18 | D | Light orange |
| ≥ 19 | E | Dark orange |

| **Nutri-Score total points for fats, oils, nuts and seeds^[[2]](#footnote-3)^** | | | | | | | |
| --- | --- | --- | --- | --- | --- | --- | --- |
| Points | Unfavorable components | | | | Favorable components | | |
|  | Energy from saturated fat  (kJ per 100g)* | Sugars  (g per 100g) | Saturated fat/total fat (%) | Salt  (g per 100g) | Protein (g per 100g) | Fiber (g per 100g) | Fruit, vegetables, legumes (%) |
| 0 | ≤ 120 | ≤ 3.4 | < 10 | ≤ 0.2 | ≤ 2.4 | ≤ 3.0 | ≤ 40 |
| 1 | > 120 | > 3.4 | < 16 | > 0.2 | > 2.4 | > 3.0 | > 40 |
| 2 | > 240 | > 6.8 | < 22 | > 0.4 | > 4.8 | > 4.1 | > 60 |
| 3 | > 360 | > 10 | < 28 | > 0.6 | > 7.2 | > 5.2 | - |
| 4 | > 480 | > 14 | < 34 | > 0.8 | > 9.6 | > 6.3 | - |
| 5 | > 600 | > 17 | < 40 | > 1.0 | > 12 | > 7.4 | > 80 |
| 6 | > 720 | > 20 | < 46 | > 1.2 | > 14 |  |  |
| 7 | > 840 | > 24 | < 52 | > 1.4 | > 17 |  |  |
| 8 | > 960 | > 27 | < 58 | > 1.6 |  |  |  |
| 9 | > 1080 | > 31 | < 64 | > 1.8 |  |  |  |
| 10 | > 1200 | > 34 | ≥ 64 | > 2.0 |  |  |  |
| 11 |  | > 37 |  | > 2.2 |  |  |  |
| 12 |  | > 41 |  | > 2.4 |  |  |  |
| 13 |  | > 44 |  | > 2.6 |  |  |  |
| 14 |  | > 48 |  | > 2.8 |  |  |  |
| 15 |  | > 51 |  | > 3.0 |  |  |  |
| 16 |  |  |  | > 3.2 |  |  |  |
| 17 |  |  |  | > 3.4 |  |  |  |
| 18 |  |  |  | > 3.6 |  |  |  |
| 19 |  |  |  | > 3.8 |  |  |  |
| 20 |  |  |  | > 4.0 |  |  |  |
| *Energy from saturated fat = saturated fat (g per 100 grams) x 37 | | | | | | | |

**Nutri-Score for fats, oils, nuts and seeds calculation:**

Summarize points from unfavorable components.

If unfavorable component is ≥ 7 points, then apply formula:
Nutri-Score total points = total unfavorable components points - (points from the fiber component + points from the fruit, vegetables and legumes component)

If unfavorable component is < 7 points, then apply formula:
Nutri-Score total points = total unfavorable component points – total favorable components points

Classify Nutri-Score using the table below.

| Nutri-Score total points for fats, oils, nuts and seeds | Class | Color |
| --- | --- | --- |
| ≤ -6 | A | Dark green |
| -5 to 2 | B | Light green |
| 3 to 10 | C | Yellow |
| 11 to 18 | D | Light orange |
| ≥ 19 | E | Dark orange |

| **Nutri-Score total points for beverages^[[3]](#footnote-4)^** | | | | | | | | |
| --- | --- | --- | --- | --- | --- | --- | --- | --- |
| Points | Unfavorable components | | | | | Favorable components | | |
|  | Energy (kJ per 100ml) | Sugars (g per 100ml) | Saturated fat (g per 100ml) | Salt  (g per 100ml) | Non-nutritive sweeteners  (absence/presence) | Protein (g per 100ml) | Fiber  (g per 100ml) | Fruit, vegetables, legumes (%) |
| 0 | ≤ 30 | ≤ 0.5 | ≤ 1.0 | ≤ 0.2 |  | ≤ 1.2 | ≤ 3.0 | ≤ 40 |
| 1 | ≤ 90 | ≤ 2.0 | > 1.0 | > 0.2 |  | > 1.2 | > 3.0 | - |
| 2 | ≤ 150 | ≤ 3.5 | > 2.0 | > 0.4 |  | > 1.5 | > 4.1 | > 40 |
| 3 | ≤ 210 | ≤ 5.0 | > 3.0 | > 0.6 |  | > 1.8 | > 5.2 | - |
| 4 | ≤ 240 | ≤ 6.0 | > 4.0 | > 0.8 | Presence | > 2.1 | > 6.3 | > 60 |
| 5 | ≤ 270 | ≤ 7.0 | > 5.0 | > 1.0 |  | > 2.4 | > 7.4 | - |
| 6 | ≤ 300 | ≤ 8.0 | > 6.0 | > 1.2 |  | > 2.7 |  | > 80 |
| 7 | ≤ 330 | ≤ 9.0 | > 7.0 | > 1.4 |  | > 3.0 |  |  |
| 8 | ≤ 360 | ≤ 10 | > 8.0 | > 1.6 |  |  |  |  |
| 9 | ≤ 390 | ≤ 11 | > 9.0 | > 1.8 |  |  |  |  |
| 10 | > 390 | > 11 | > 10 | > 2.0 |  |  |  |  |
| 11 |  |  |  | > 2.2 |  |  |  |  |
| 12 |  |  |  | > 2.4 |  |  |  |  |
| 13 |  |  |  | > 2.6 |  |  |  |  |
| 14 |  |  |  | > 2.8 |  |  |  |  |
| 15 |  |  |  | > 3.0 |  |  |  |  |
| 16 |  |  |  | > 3.2 |  |  |  |  |
| 17 |  |  |  | > 3.4 |  |  |  |  |
| 18 |  |  |  | > 3.6 |  |  |  |  |
| 19 |  |  |  | > 3.8 |  |  |  |  |
| 20 |  |  |  | > 4.0 |  |  |  |  |

**Nutri-Score for beverages calculation:**

Summarize points from unfavorable components.

Summarize points from favorable components.

Nutri-Score total points = total unfavorable component points – total favorable components points

Classify Nutri-Score using the table below.

| Nutri-Score total points for beverages | Class | Color |
| --- | --- | --- |
| Water* | A | Dark green |
| ≤ 2 | B | Light green |
| 3 to 6 | C | Yellow |
| 7 to 9 | D | Light orange |
| ≥ 10 | E | Dark orange |
| *Plain water was automatically given Nutri-Score class A. | | |

The algorithms can be found in full in the “Update report from the Scientific Committee of the Nutri-Score 2022” page 130-135^[[4]](#footnote-5)^ and “Update of the Nutri-Score algorithm for beverages (2023)” page 72-75^[[5]](#footnote-6)^.

1. The Scientific Committee of the Nutri-Score. Update of the Nutri-Score algorithm. Update report from the Scientific Committee of the Nutri-Score 2022. Santé Publique France; 2022. [↑](#footnote-ref-2)
2. The Scientific Committee of the Nutri-Score. Update of the Nutri-Score algorithm. Update report from the Scientific Committee of the Nutri-Score 2022. Santé Publique France; 2022. [↑](#footnote-ref-3)
3. The Scientific Committee of Nutri-Score. Update of the Nutri-Score algorithm for beverages. Second update report from the Scientific Committee of the Nutri-Score V2 – 2023. Santé Publique France; 2023. [↑](#footnote-ref-4)
4. The Scientific Committee of the Nutri-Score. Update of the Nutri-Score algorithm. Update report from the Scientific Committee of the Nutri-Score 2022. Santé Publique France; 2022. [↑](#footnote-ref-5)
5. The Scientific Committee of Nutri-Score. Update of the Nutri-Score algorithm for beverages. Second update report from the Scientific Committee of the Nutri-Score V2 – 2023. Santé Publique France; 2023. [↑](#footnote-ref-6)
