# Supplementary material for: How does the updated Nutri-Score discriminate and classify the nutritional quality of foods in a Norwegian setting?
Source: Int J Behav Nutr Phys Act. 2023 Oct 10;20:122. doi: 10.1186/s12966-023-01525-y (PMC10563306; doi:10.1186/s12966-023-01525-y)
Supplement: Supplementary file 2 — Additional file 2. Main food categories. [file 12966_2023_1525_MOESM2_ESM.docx]

**Additional file 2. Main food categories**

Description of content in each main category of food and beverages:

- *Fruit, vegetables, legumes, potatoes, and products thereof:* fresh, frozen, dried, and canned fruits, vegetables and legumes; potatoes; potato-products except crisps, vegetable spreads; vegetables mixes; tomato puree; pickled veggies; plant-based meat alternatives; tofu; jams; and fruit compotes.
- *Cereals, grains and similar, and products thereof:* flours; grains/cereals including couscous and quinoa; breakfast cereals; rice; pasta; various types of breads including crisp breads, rolls, baguettes, ciabatta, etc.; noodles; tortillas; crackers; and sweet bakery products such as biscuits, cookies, pastries, cakes including bake mixes.
- *Fish, meat, eggs, and products thereof:* raw meat of poultry, hare, pork, cow, lamb, sheep, and game; fish and seafood; meat and fish spreads and cold cuts; minced meat; breaded meat and fish; sausages; meat balls; burgers; fish cakes; and eggs.
- *Dairy products:* yoghurt; quark; cheese; ice cream; and dairy desserts such as pudding, mousse. Creams and cooking fats are not included in this category but included in a separate category for fats and oils due to the Nutri-Score algorithm.
- *Sauces and dressings:* sauces; ketchup; mustard; paste; mayonnaise; dressing; and pesto. Both prepared and dry powder mixes.
- *Sweet and/or salty snacks:* sweet spreads; chocolate; candy; honey; jelly; drops; sugar; sirup; crisps; popcorn; tortilla chips; and cheese puff snacks.
- *Composite foods and meals:* plant-based cheese and yoghurt alternatives; salads used as spreads; and semi-ready and ready meals, such as pasta meals (including lasagna), sandwiches, soups, pizza, pies, stews, porridge, and other ready meals (e.g., fish au gratin, salads). Both prepared and dry powder/package mixes.
- *Fats and oils:* butter; margarine; cooking fats; oils; and creams (e.g., creams, crème fraiche, sour cream, and plant-based cream options including coconut milk).
- *Nuts and seeds:* unsalted and salted nuts and seeds; nut mixes; and nut/seed products with more than 50% nuts/seeds, such as tahini, peanut butter, and coated nuts.
- *Plain water:* plain water.
- *Water-based beverages:* soda; squash; energy drinks; sport drinks; iced teas; coffee; tea; flavored and carbonated water; and non-alcoholic beer and wine. Beverages could be with and without added sugar and non-nutritive sweeteners.
- *Fruit- and vegetable-based beverages:* fruit and vegetable juices; nectars; and smoothies. Beverages could be with and without added sugar and non-nutritive sweeteners.
- *Dairy beverages and plant-based alternatives:* milks; fermented milks; flavored milks; and plant-based milk alternatives with and without fortification of nutrients. Beverages could be with and without added sugar and non-nutritive sweeteners.
