# Supplementary material for: How does the updated Nutri-Score discriminate and classify the nutritional quality of foods in a Norwegian setting?
Source: Int J Behav Nutr Phys Act. 2023 Oct 10;20:122. doi: 10.1186/s12966-023-01525-y (PMC10563306; doi:10.1186/s12966-023-01525-y)
Supplement: Supplementary file 3 — Additional file 3. Subcategories of foods. [file 12966_2023_1525_MOESM3_ESM.docx]

**Additional file 3. Subcategories of foods**

Detailed description of content in each subcategory of food and beverages:

- *Fruit, vegetables, and legumes*: raw, canned, frozen, dried fruits, vegetables and legumes.
- *Potatoes and potato products*: raw and boiled potatoes; potato salads; fried potatoes; potatoes au gratin; and mashed potatoes. Not including potato crisps.
- *Grains, pasta, rice, noodles*: grains; quinoa; rice; pasta; noodles; and couscous. Mostly dried products, but also a few prepared.
- *Flours, flour mixes*: various flours and flour mixes, including those of legumes and coconut.
- *Breads*: regular and crisp bread; baguettes; rolls; thin wafer crispbread; ciabatta; croutes; breads for burgers and sausages; and naan.
- *Breakfast cereals*: various types of breakfast cereals and grains.
- *Eggs*: eggs, including whites and yolk.
- *Fish and seafood*: raw, frozen, dried, salted, and smoked fish and seafood; fish patties; cured fish; and breaded fish. Excluding typical fish spreads and sandwich toppings included in the sandwich toppings category.
- *Meat – red:* both unprocessed and processed meat of pork, cow, game, lamb, sheep, and game, such as raw, salted, smoked, and dried meat; meatballs; meat patties; sausages; and minced meat. Excluding typical cold cuts and meat spreads included in the sandwich toppings category.
- *Meat – poultry:* both unprocessed and processed meat of poultry, mainly turkey and chicken (but also hen, duck). For example, raw meat; meatballs; meat patties; sausages; and minced meat. Excluding typical cold cuts and meat spreads included in the sandwich toppings category.
- *Plant-based meat alternatives*: plant-based balls; sausages; minced meat alternatives; burgers; falafel; and tofu. Excluding typical alternatives used as spreads included in the sandwich toppings category
- *Yoghurt and plant-based alternatives*: quark; yoghurts; and yoghurt with muesli. Includes plant-based yoghurt imitates.
- *Cheese and plant-based alternatives*: various types of cheeses, including brown cheese. Includes plant-based cheese imitates.
- *Sandwich toppings:* jams; mayo-based salads used as spreads; fish- and meat-based spreads and cold cuts; plant-based spreads such as vegetable pastes, spreads from nuts and nougat. Excluding cheese which is a separate category.
- *Sauces, dressings:* sauces; ketchup; mustard; paste; mayonnaise; dressing; and pesto. Include both prepared and dry powder mixes.
- *Crisps*: crisps; popcorn; tortilla chips; and cheese puff snacks.
- *Chocolate, candy*: chocolate; candy; and drops.
- *Sweet biscuits/pastries:* sweet cookies/crackers/biscuits; and sweet pastries.
- *Desserts, cakes, and ice cream*: desserts such as mousse and pudding; dessert sauces; jelly; cakes including bake mixes, waffles, muffins; compotes; and ice creams.
- *Ready meals*: meals that are ready to eat or just need reheating, such as prepared or canned soups and stews; composite meals; pizza; pies; fish au gratin; pasta dishes including lasagna; and (rice) porridge.
- *Semi-ready meals*: soups; casseroles; and meals that need some preparation. Include dry powder/package mixes and as prepared.
- *Miscellaneous*: for general foods, products that were too few to gather in their own category or hard to place were included in the miscellaneous group, such as salted crackers/biscuits; tortillas/wraps/chapatti/thin potato griddle; other types of meat (rabbit, hare, whale that are – not typically red or poultry); and sugar/honey.
- *Oils and soft/liquid margarine*: oils and soft/liquid margarine with <25 grams saturated fat per 100 grams, such as olive oils and soft margarines. This threshold is specified in the Norwegian food-based dietary guidelines under the recommendation to choose edible liquid oils, liquid margarine and soft margarine spreads instead of hard margarines and butter^[[1]](#footnote-2)^.
- *Butterblends and hard margarine*: butterblends and hard margarine with 25-50 grams saturated fat per 100 grams, such as hard margarines; palm oil; and butter blends. This threshold is specified in the Norwegian food-based dietary guidelines under the recommendation to choose edible liquid oils, liquid margarine and soft margarine spreads instead of hard margarines and butter^[[2]](#footnote-3)^.
- *Butter and hard oils*: butter and hard oils with >50 grams saturated fat per 100 grams, such as butter and coconut oil. This threshold is specified in the Norwegian food-based dietary guidelines under the recommendation to choose edible liquid oils, liquid margarine and soft margarine spreads instead of hard margarines and butter^[[3]](#footnote-4)^.
- *Cremes*: cremes used for cooking, such as cooking creams; crème fraiche; sour cream; including plant-based cream alternatives and coconut milks used for cooking.
- *Unsalted nuts*: unsalted nuts, such as pecan, peanut, cashew, etc.
- *Salted/coated nuts*: salted nuts (peanut, cashew, etc.); nut mixes; chili nuts; and peanut butter.
- *Seeds*: plain seeds (sunflower seeds, poppy, etc.) and tahini.
- *Plain water:* plain water.
- *Other*: coffee; tea (with and without sweeteners); carbonated water; flavored water; and non-alcoholic beer and wine.
- *Sugar-sweetened beverages*: beverages with added sugar, such as soda, squash, kombucha, lemonade, energy drinks, mulled claret, iced tea, and sport drinks.
- *Artificially sweetened beverages*: beverages with non-nutritive sweeteners (no added sugars) such as soda, iced tea, energy drinks, and squash.
- *Fruit- and vegetable-based beverages*: fruit and vegetable juices; smoothies; and nectars. Beverages could be with and without added sugar and non-nutritive sweeteners.
- *Milk and dairy-based beverages:* milk (skimmed, partly skimmed, whole fat); fermented milks; milk with flavors; cocoa; protein shakes; and iced coffee. Beverages could be with and without added sugar and non-nutritive sweeteners.
- *Plant-based beverages*: plant-based milk alternatives (e.g., beverages from almond, soy, rice, oats, and coconut), including options that are flavored and fortified. Beverages could be with and without added sugar and non-nutritive sweeteners.

1. Helsedirektoratet. Kostråd om margarin, olje og smør- Helsenorge: Norsk helsenett SF; 2022 [cited 2023 June 27]. Available from: https://www.helsenorge.no/kosthold-og-ernaring/kostrad/velg-matoljer-og-myk-margarin/. [↑](#footnote-ref-2)
2. Helsedirektoratet. Kostråd om margarin, olje og smør- Helsenorge: Norsk helsenett SF; 2022 [cited 2023 June 27]. Available from: https://www.helsenorge.no/kosthold-og-ernaring/kostrad/velg-matoljer-og-myk-margarin/. [↑](#footnote-ref-3)
3. Helsedirektoratet. Kostråd om margarin, olje og smør- Helsenorge: Norsk helsenett SF; 2022 [cited 2023 June 27]. Available from: https://www.helsenorge.no/kosthold-og-ernaring/kostrad/velg-matoljer-og-myk-margarin/. [↑](#footnote-ref-4)
