# Supplementary material for: How does the updated Nutri-Score discriminate and classify the nutritional quality of foods in a Norwegian setting?
Source: Int J Behav Nutr Phys Act. 2023 Oct 10;20:122. doi: 10.1186/s12966-023-01525-y (PMC10563306; doi:10.1186/s12966-023-01525-y)
Supplement: Supplementary file 4 — Additional file 4. The development of the Norwegian food-based dietary guidelines. [file 12966_2023_1525_MOESM4_ESM.docx]

**Additional file 4. The development of the Norwegian food-based dietary guidelines.**

The methodology and scientific background for the Norwegian food-based dietary guidelines (FBDGs) are described in a 350-page report by the Norwegian Nutrition Council from 2011^[[1]](#footnote-1)^. Unfortunately, the report is only published in Norwegian language.

In brief, the current Norwegian FBDGs were developed from a review of systematic reviews (available until December 2010) and evaluation of the quality of evidence of the association of foods with obesity and chronic diet-related diseases, with the aim to prevent the aforementioned diet-related diseases in the Norwegian population^[[2]](#footnote-2)^. The systematic reviews evaluated in the development of the Norwegian FBDGs have been conducted by other, independent organizations and health authorities. Only evidence deemed convincing or probable was strong enough to make the basis for the official recommendations. The methodology was in line with how the World Cancer Research Fund drew conclusions from the research literature in 2007.

As evidence-based reviews rarely form concrete recommendations, an overall assessment of several diseases, foods and physical activity was taken into account in the development of the Norwegian FBDGs^[[3]](#footnote-3)^. Research on foods’ effect on risk of disease was the primary basis for the recommendations if the research documented a convincing or probably causal relationship between exposure (food) and disease. Research and knowledge on nutrients were used if research on foods were limited or lacking. If a food group was a good source of a nutrient (i.e., >20% of a nutrient comes from a specific food category in an average Norwegian diet, such as fish is for vitamin D), nutrient-based research was also included in the assessment. Often, a combination of results from food-based and nutrient-based research was used in the development of the FBDGs. The report describing the methodology and scientific background for the Norwegian FBDGs also mentioned that to provide recommendations to be used by the population, the recommendations should be concrete and easy to understand. Therefore, the recommendations are quantitative if the evidence was deemed sufficient, based on convincing or probable causal associations.

1. Helsedirektoratet. Kostråd for å fremme folkehelsen og forebygge kroniske sykdommer. Metodologi og vitenskapelig kunnskapsgrunnlag. 2011. Report No.: IS-1881. [↑](#footnote-ref-1)
2. Helsedirektoratet. Kostråd for å fremme folkehelsen og forebygge kroniske sykdommer. Metodologi og vitenskapelig kunnskapsgrunnlag. 2011. Report No.: IS-1881. [↑](#footnote-ref-2)
3. Helsedirektoratet. Kostråd for å fremme folkehelsen og forebygge kroniske sykdommer. Metodologi og vitenskapelig kunnskapsgrunnlag. 2011. Report No.: IS-1881. [↑](#footnote-ref-3)
