# Supplementary material for: How does the updated Nutri-Score discriminate and classify the nutritional quality of foods in a Norwegian setting?
Source: Int J Behav Nutr Phys Act. 2023 Oct 10;20:122. doi: 10.1186/s12966-023-01525-y (PMC10563306; doi:10.1186/s12966-023-01525-y)
Supplement: Supplementary file 5 — Additional file 5. Distribution of Nutri-Score classes for main food categories. [file 12966_2023_1525_MOESM5_ESM.docx]

**Additional file 5. Distribution of Nutri-Score classes for main food categories.**

| **Table**. Distribution of Nutri-Score classes for main food categories in a Norwegian food database. | | | | | | |
| --- | --- | --- | --- | --- | --- | --- |
|  | Nutri-Score class | | | | | Total |
|  | A | B | C | D | E |  |
| Fruit, vegetables, legumes, potatoes and products thereof | 64.5  (209) | 5.9  (19) | 20.1  (65) | 7.7  (25) | 1.9  (6) | 100.0 (324) |
| Cereals, grains and products thereof | 30.8  (102) | 16.9  (56) | 24.5  (81) | 15.7  (52) | 12.1  (40) | 100.0 (331) |
| Fish, meat, eggs and products thereof | 33.6  (120) | 7.3  (26) | 14.9  (53) | 28.9 (103) | 15.4  (55) | 100.0 (357) |
| Dairy products* | 10.4  (17) | 7.3  (12) | 25.6  (42) | 38.4  (63) | 18.3  (30) | 100.0 (164) |
| Sauces, dressings | 4.4  (3) | 2.9  (2) | 30.9  (21) | 29.4  (20) | 32.4  (22) | 100.0 (68) |
| Sweet and/or salty snacks | 3.6  (4) | 2.7  (3) | 8.9  (10) | 18.8  (21) | 66.1  (74) | 100.0 (112) |
| Composite foods, meals | 5.4  (6) | 17.9  (20) | 44.6  (50) | 21.4  (24) | 10.7  (12) | 100.0 (112) |
| Fats, oils | 0.0  (0) | 15.7  (11) | 27.1  (19) | 37.1  (26) | 20.0  (14) | 100.0 (70) |
| Nuts, seeds | 54.3  (19) | 17.1  (6) | 17.1  (6) | 11.4  (4) | 0.0  (0) | 100.0 (35) |
| Plain water** | 100.0  (3) | 0.0  (0) | 0.0  (0) | 0.0  (0) | 0.0  (0) | 100.0  (3) |
| Water-based beverages | NA | 24.7  (23) | 29.0  (27) | 14.0  (13) | 32.3  (30) | 100.0 (93) |
| Fruit and vegetable-based beverages | NA | 7.7  (3) | 48.7  (19) | 28.2  (11) | 15.4  (6) | 100.0 (39) |
| Dairy beverages and plant-based alternatives | NA | 35.1  (26) | 32.4  (24) | 17.6  (13) | 14.9  (11) | 100.0 (74) |
| Total | 27.1  (483) | 11.6 (207) | 23.4 (417) | 21.0 (375) | 16.8 (300) | 100.0 (1782) |
| Data shown as percentages (n).  *excluding cremes with are included in the fats and oils category.  **Only plain water can obtain Nutri-Score A for beverages.  NA: Not applicable. | | | | | | |
