# Supplementary material for: How does the updated Nutri-Score discriminate and classify the nutritional quality of foods in a Norwegian setting?
Source: Int J Behav Nutr Phys Act. 2023 Oct 10;20:122. doi: 10.1186/s12966-023-01525-y (PMC10563306; doi:10.1186/s12966-023-01525-y)
Supplement: Supplementary file 6 — Additional file 6. Distribution of Nutri-Score classes for subcategories of general foods. [file 12966_2023_1525_MOESM6_ESM.docx]

**Additional file 6. Distribution of Nutri-Score classes for subcategories of general foods**

| **Table**. Distribution of Nutri-Score classes for subcategories of foods. | | | | | | |
| --- | --- | --- | --- | --- | --- | --- |
|  | Nutri-Score class | | | | | Total |
|  | A | B | C | D | E |  |
| **General foods** | | | | | | |
| Fruit, vegetables, legumes | 79.8 (186) | 4.7 (11) | 9.0 (21) | 4.3 (10) | 2.2 (5) | 100.0 (233) |
| Potatoes and potato products | 27.3 (6) | 18.2 (4) | 50.0 (11) | 4.6 (1) | 0.0 (0) | 100.0 (22) |
| Grains, pasta, rice, noodles | 51.1 (23) | 33.3 (15) | 8.9 (4) | 4.4 (2) | 2.2 (1) | 100.0 (45) |
| Flour, flour mixes | 68.4 (26) | 2.6 (1) | 21.1 (8) | 7.9 (3) | 0.0 (0) | 100.0 (38) |
| Breads | 32.7 (36) | 24.6 (27) | 34.6 (38) | 8.2 (9) | 0.0 (0) | 100.0 (110) |
| Breakfast cereals | 33.3 (12) | 16.7 (6) | 30.6 (11) | 11.1 (4) | 8.3 (3) | 100.0 (36) |
| Eggs | 80.0 (4) | 0.0 (0) | 0.0 (0) | 20.0 (1) | 0.0 (0) | 100.0  (5) |
| Fish, seafood* | 54.4 (56) | 10.7 (11) | 17.5 (18) | 12.6 (13) | 4.9 (5) | 100.0 (103) |
| Meat – red* | 21.6 (30) | 7.2 (10) | 18.7 (26) | 33.1 (46) | 19.4 (27) | 100.0 (139) |
| Meat – poultry* | 55.0 (22) | 5.0 (2) | 2.5 (1) | 37.5 (15) | 0.0 (0) | 100.0 (40) |
| Plant-based meat alternatives* | 41.7 (10) | 12.5 (3) | 25.0 (6) | 20.8 (5) | 0.0 (0) | 100.0 (24) |
| Yoghurt and plant-based alternatives | 29.6 (13) | 25.0 (11) | 43.2 (19) | 2.3 (1) | 0.0 (0) | 100.0 (44) |
| Cheese and plant-based alternatives | 3.6 (3) | 0.0 (0) | 11.9 (10) | 58.3 (49) | 26.2 (22) | 100.0 (84) |
| Sandwich toppings | 8.3 (10) | 3.3 (4) | 28.9 (35) | 37.2 (45) | 22.3 (27) | 100.0 (121) |
| Sauces, dressings | 4.4 (3) | 2.9 (2) | 30.9 (21) | 29.4 (20) | 32.4 (22) | 100.0 (68) |
| Crisps | 6.3 (1) | 6.3 (1) | 6.3 (1) | 43.8 (7) | 37.5 (6) | 100.0 (16) |
| Chocolate, candy | 1.7 (1) | 0.0 (0) | 0.0 (0) | 6.8 (4) | 91.5 (54) | 100.0 (59) |
| Sweet biscuits/pastries | 0.0 (0) | 0.0 (0) | 11.1 (5) | 46.7 (21) | 42.2 (19) | 100.0 (45) |
| Desserts, cakes, ice cream | 3.3 (3) | 5.4 (5) | 29.4 (27) | 31.5 (29) | 30.4 (28) | 100.0 (92) |
| Ready meals | 8.5 (5) | 23.7 (14) | 39.0 (23) | 28.8 (17) | 0.0 (0) | 100.0 (59) |
| Semi-ready meals | 2.4 (1) | 16.7 (7) | 57.1 (24) | 2.4 (1) | 21.4 (9) | 100.0 (42) |
| Miscellaneous | 23.3 (10) | 9.3 (4) | 30.2 (13) | 11.6 (5) | 25.6 (11) | 100.0 (43) |
| **Fats, oils, nuts and seeds** | | | | | | |
| Oils and soft/liquid margarine | 0.0 (0) | 35.5 (11) | 51.6 (16) | 12.9 (4) | 0.0 (0) | 100.0 (31) |
| Butterblends and hard margarine | 0.0 (0) | 0.0 (0) | 0.0 (0) | 40.0 (4) | 60.0 (6) | 100.0 (10) |
| Butter and hard oils | 0.0 (0) | 0.0 (0) | 0.0 (0) | 0.0 (0) | 100.0 (8) | 100.0  (8) |
| Cremes** | 0.0 (0) | 0.0 (0) | 14.3 (3) | 85.7 (18) | 0.0 (0) | 100.0 (21) |
| Unsalted nuts | 81.8 (9) | 18.2 (2) | 0.0 (0) | 0.0 (0) | 0.0 (0) | 100.0 (11) |
| Salted/coated nuts | 0.0 (0) | 23.1 (3) | 46.2 (6) | 30.8 (4) | 0.0 (0) | 100.0 (13) |
| Seeds | 90.9 (10) | 9.1 (1) | 0.0 (0) | 0.0 (0) | 0.0 (0) | 100.0 (11) |
| **Beverages** | | | | | | |
| Plain water*** | 100.0 (3) | 0.0 (0) | 0.0 (0) | 0.0 (0) | 0.0 (0) | 100.0  (3) |
| Other beverages**** | 0.0 (0) | 82.1 (23) | 14.3 (4) | 3.6 (1) | 0.0 (0) | 100.0 (28) |
| Sugar-sweetened beverages | 0.0 (0) | 0.0 (0) | 6.8 (3) | 27.3 (12) | 65.9 (29) | 100.0 (44) |
| Artificially sweetened beverages | 0.0 (0) | 0.0 (0) | 95.2 (20) | 0.0 (0) | 4.8 (1) | 100.0 (21) |
| Fruit and vegetable-based beverages | 0.0 (0) | 7.7 (3) | 48.7 (19) | 28.2 (11) | 15.4 (6) | 100.0 (39) |
| Milk and dairy-based beverages | 0.0 (0) | 38.2 (21) | 34.6 (19) | 10.9 (6) | 16.4 (9) | 100.0 (55) |
| Plant-based beverages | 0.0 (0) | 26.3 (5) | 26.3 (5) | 36.8 (7) | 10.5 (2) | 100.0 (19) |
| Total | 27.1 (483) | 11.6 (207) | 23.4 (417) | 21.0 (375) | 16.8 (300) | 100.0 (1782) |
| Data shown as percentages (n).  *Excluding typical sandwich spreads and cold cuts used as sandwich toppings which are included in the sandwich toppings category.  **Creams: used for cooking, such as regular cream, crème fraiche, sour cream, and plant-based cream alternatives.  ***Plain water is automatically given Nutri-Score class A.  ****Other beverages include carbonated and flavored water, tea, coffee, and non-alcoholic wine/beer. | | | | | | |
