# Supplementary material for: How does the updated Nutri-Score discriminate and classify the nutritional quality of foods in a Norwegian setting?
Source: Int J Behav Nutr Phys Act. 2023 Oct 10;20:122. doi: 10.1186/s12966-023-01525-y (PMC10563306; doi:10.1186/s12966-023-01525-y)
Supplement: Supplementary file 7 — Additional file 7. Distribution of Nutri-Score for main categories of foods. [file 12966_2023_1525_MOESM7_ESM.docx]

**Additional file 7. Distribution of Nutri-Score for main categories of foods**

**
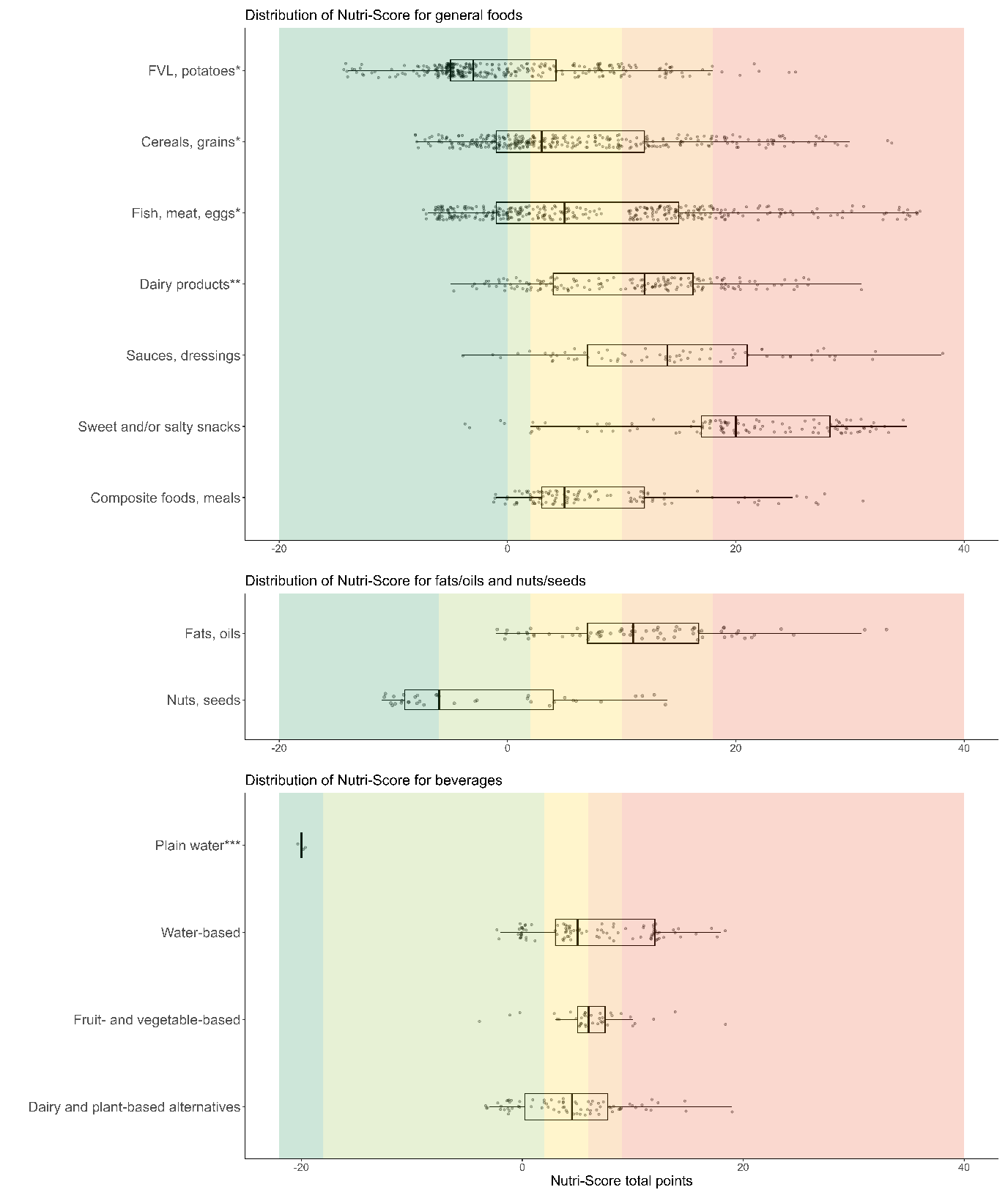
Figure.** Distribution of Nutri-Score for main categories of general foods (top), nuts/seeds and fats/oils (middle) and beverages (bottom).
Distributions are shown with boxplots where the vertical line in the box represent the median total points, the box indicate the 25^th^ and 75^th^ percentile and the whiskers indicate the lowest or highest value (maximum higher or lower than 1.5 x the interquartile range). The dots represent all single products within the category. Dark green color background indicates Nutri-Score class A, light green Nutri-Score class B, yellow Nutri-Score class C, light orange Nutri-Score class D, and dark orange indicates products classified with Nutri-Score E. *and products thereof; **excluding cremes which are included in the fats and oils category; ***plain water is not given total points but is included for illustrative purposes. FVL: Fruit, vegetables, and legumes.
