# Supplementary material for: How does the updated Nutri-Score discriminate and classify the nutritional quality of foods in a Norwegian setting?
Source: Int J Behav Nutr Phys Act. 2023 Oct 10;20:122. doi: 10.1186/s12966-023-01525-y (PMC10563306; doi:10.1186/s12966-023-01525-y)
Supplement: Supplementary file 8 — Additional file 8. Detailed description of the updated Nutri-Score for subcategories of foods. [file 12966_2023_1525_MOESM8_ESM.docx]

**Additional file 8. Detailed description of the updated Nutri-Score for subcategories of foods**

Only generic food names are used in the description below.

- **Fruit, vegetables and legumes** (n = 233): Most (80%) of foods in the fruit, vegetables and legumes category were classified with Nutri-Score A and those were mainly raw or unprocessed foods. Fruit, vegetables and legumes that got classified with Nutri-Score D or E had a higher energy (kJ) and/or sugar and/or salt content and/or lower fruit, vegetable and legume content (such as olives in oil, dried banana/papaya) or were in powder forms (turnip stew). Foods in this category classified with Nutri-Score C were mostly pickled or in sugar brine.
- **Potatoes and potato products** (n = 22): Raw and boiled potatoes were classified with Nutri-Score A or B. In general, more processed potatoes and potato products were classified with Nutri-Score C or D, but most products were classified with Nutri-Score C (50%). Frozen french fries got Nutri-Score A (total points of -1), likely because it is low in salt and higher in fiber. Raw sweet potato was classified with Nutri-Score B (2 total points) and was lower in fiber and protein compared to the frozen french fries. Potato salads all got Nutri-Score C, but total points varied: Potato salad with crème fraiche got 9 total points, low-fat sour cream got 6 total points, potato salad with regular sour cream got 8 total points, potato salad with full-fat mayonnaise got 9 total points and potato salad with light mayonnaise also got 9 total points. The differences between the potato salads seemed to get pick-up in the Nutri-Score total points by kJ, salt and saturated fat content, but not by the Nutri-Score class.
- **Grains, pasta, rice, noodles** (n = 45): Over 80% of products in this category were classified with Nutri-Score A or B. Fresh pasta got Nutri-Score B (total points: 2) and refined pasta dry got A (-2 total points), pasta plates dry A (-2 total points), spaghetti dry A (-2 total points), pasta with beans A (-6 total points), and wholegrain pasta dry A (-5 total points). Wild rice dry got A (-4 total points), whole grain rice dry got A (-1 total points), Basmati rice dry B (1 total points), wild rice mix dry B (1 total points), Jasmine rice dry B (1 total points), and quick-to-boil rice dry B (1 total points). Several cereals (couscous, quinoa, semolina, oatmeal, barley) were classified with Nutri-Score A. Noodles in a bag got E. Overall, this may suggest that the total points captured the differences between the products, but not the Nutri-Score classes as most refined and whole grain pasta were classified with Nutri-Score A.
- **Flours, flour mixes** (n = 38): Most products got Nutri-Score A (70%). Gluten-free products more often got a Nutri-Score C, but one bread flour mix got a D (likely because the salt content in the latter product was high). Coarse spelt flour got A (-8 total points) and fine spelt flour Nutri-Score A (-3 total points). Wholemeal wheat flour got A (-6 total points), while sifted wheat flour also obtained Nutri-Score A (-3 total points). Similarly for wholemeal rye flour and sifted rye flour. Overall, this suggest that the Nutri-Score total points (not the Nutri-Score class) captured the differences.
- **Breads** (n = 110): There were breads in all Nutri-Score classes except for E, most were classified with Nutri-Score A (33%) or C (35%). One crispbread got D (11 total points), likely because it had a higher fat content than other breads, which was captured by the kJ component in Nutri-Score algorithm. White breads were typically classified with Nutri-Score C but could also get a Nutri-Score B.
- **Breakfast cereals** (n = 36): There were breakfast cereals in all Nutri-Score classes, most were categorized with Nutri-Score class A (33%). Many (17 av 36) breakfast cereals had a fiber content above 7.4 g (max point from the fiber component in the Nutri-Score algorithm), thus fiber content above this is not differentiated in the algorithm. Breakfast cereals with the most sugar got poorer Nutri-Score classes. Still, one breakfast cereal with 19g of sugar per 100 grams got Nutri-Score B (1 total points), possibly due to relatively high fiber and protein content, and that it is lower in energy. Two products with 11 grams of sugar per 100 grams got Nutri-Score A, likely due to the high fiber content. The most unhealthy (highest sugar and salt content) got D or E. One of the fruit muesli’s was classified with Nutri-Score C (10 total points), possibly due to the sugar content from dried fruit and lower fiber content.
- **Eggs** (n = 5): Eggs and egg whites were classified with Nutri-Score A (Nutri-Score total points of -1 and -2 respectively), while yolk got D (11p).
- **Fish and seafood** (n = 103): Most fish products were classified with Nutri-Score A (54%). Breaded coalfish were classified with Nutri-Score A, while raw, unprocessed fish got As. Cured halibut was classified with Nutri-Score B (2 total points). Cured salmon got E (19 total points), various mixed fish products ranged from B to D, such as fish balls (B and C), fish sticks (Nutri-Score B), fish cakes got Cs and Ds, and fish patties got B, C and Ds. Salmon fish patties got B. Farmed (raw) salmon got Nutri-Score A (-3 total points). Products that were classified with Nutri-Score E were more processed: salted or cured (high in salt).
- **Red – meat** (n = 139): 22% of red meat products were classified with Nutri-Score A, and those products were mainly raw, unprocessed red meat – often the lean meats e.g. beef and pork sirloin were classified with Nutri-Score A, both got -1 total points. While raw red meats with more fat more often got classified with Nutri-Score C (e.g. rib eye/entrecôte, 5 total points). More processed meat products were mostly classified with Nutri-Score D or E. Leaner sausages got Ds (13 total points), and regular sausage options got Nutri-Score D or E (16/18 or 19 total points). The Nutri-Score classes for sausages indicated that the updated Nutri-Score does not consistently capture the differences in fat between regular and leaner sausages, as other components affect the Nutri-Score class. Regular minced meat without water and salt was classified with Nutri-Score C (6 total points), and minced meat with less fat B (1 total points). Smoked meat also got classified with Nutri-Score Ds and Es, minced meat with less fat and with salt got C (6 total points), while regular minced meat with salt and water got D (13 total points). A notable distinction was that Nutri-Score class game meat as red meat, whereas the Norwegian health directorate does not classify game meat as red meat.
- **Meat – poultry** (n = 40): Over 50% of products in this category were classified with Nutri-Score A, but many also with a D (38%). In general, less processed and leaner poultry got better Nutri-Score classes than more processed and poultry with higher fat content. Chicken sausages mostly got Nutri-Score D, while unprocessed chicken products got classified with Nutri-Score A. Chicken mince got Nutri-Score A (-4 total points), and chicken meatballs C or D (4 and 16 total points respectively). Chicken sausages got Ds (13/16 total points). To tell the difference between sausages from red meat and poultry, one must look at the total points not the Nutri-Score class. Sausages from poultry had less saturated fat, but often more salt and these products therefore still got poorer Nutri-Scores classes (similar to sausages from red meat).
- **Plant-based meat alternatives** (n = 24): 42% of plant-based meat alternatives were classified with Nutri-Score A. One product in this category had a content of fruit, vegetables, and legume over 40% (tofu), which one might expect to be higher. The products differed in content of salt, saturated fat, energy (kJ), protein and fiber. All products were low in sugars.
- **Yogurt and plant-based alternatives** (n = 44): Most products were classified with Nutri-Score C (43%), but some also with Nutri-Score A or B, but no products in this category were classified with E and only one with D. The latter product was a yoghurt with a high content of sugar and saturated fat, which could be considered as a dessert yoghurt. One yogurt with sugar could get Nutri-Score B (2 total points), while the same yoghurt without sugar also got B (1 total points). Yoghurts classified with Nutri-Score class A were mainly low in fat and sugars and/or had a high protein content.
- **Cheese and plant-based alternatives** (n = 84): Most cheeses (58%) were classified with Nutri-Score D. Cheeses that were classified with Nutri-Score A were particular products such as “Gammelost”, an old cheese (-5 total points), probably due to low fat, carbohydrates and salt content; and cottage cheese. Semi-hard full fat cheeses (26% and 16% fat) got Nutri-Score D (13/12 total points), while similar low-fat cheeses with 10% fat got C (5 total points). In the updated Nutri-Score there is one point distinguishing the 16% and 26% fat cheeses in the total points and they were classified with the same Nutri-Score class. The updated Nutri-Score might not capture fat differences between cheeses as one might want in a Norwegian setting. However, for certain cheeses Nutri-Score captured these differences. Light cheese spreads got Nutri-Score D (11 total points), but even lighter cheese spread got C (8 total points), and regular cream cheese got D (17 total points). Light soft and sweet whey-cheese were classified with Nutri-Score E (19 total points) and regular soft and sweet whey-cheese E (22 total points); the difference in fat content was not captured, likely due to the high sugar content. Vegan cheeses got Es.
- **Sandwich toppings** (n = 121): Most sandwich toppings were classified with Nutri-Score C, D or E (30%, 38% and 22% respectively). This category consists of heterogeneous products (from meats to chocolate spreads and fish). Products classified with As were jams with a high proportion of berries and/or without sugar; tuna products, sliced roast beef, and salmon topping. Mustard herring E (24 total points) and caviar E (30 total points), which have a high salt content, were classified with Nutri- Score E, the same as cured ham, (24 total points), canned anchovies E (27 total points) and chocolate spread (24 total points). In comparison to the latter, chocolate spread without added sugars was classified with Nutri-Score C (7 total points).
- **Sauces, dressings** (n = 68): Approximately 60% of products in this category were classified with Nutri-Score D or E. There were a variety of products in this category. Products classified with Nutri-Score A in this category were low in unfavorable components, and had a higher content of vegetables, such as tomato sauce products.
- **Crisps** (n = 16): Approximately 80% of products in this category were classified with Nutri-Score D or E. Popcorn without added fat got A (-1 total points) because it has a high fiber content, some protein and is low in salt. Most products were classified with Nutri-Score D or E, 44% and 38%, respectively. The products with the highest total points (33) had a high content of energy (kJ), saturated fat and salt, even with a high protein content.
- **Chocolate, candy** (n = 59): Drops without sugar was classified with Nutri-Score A because of favorable points from fiber. Almost everything else got E (92%), some products D (7%).
- **Sweet biscuits/pastries** (n = 45): Most products were classified with either D or E, 47% and 42% respectively.
- **Dessert, cakes, ice cream** (n = 92): Products in this category were mostly classified with Nutri-Score C (30%), D (32%) or E 30%). The three products classified with Nutri-Score A were mainly high in protein and/or low in other unfavorable components.
- **Ready meals** (n = 59): Approximately 40% of ready meals were classified with Nutri-Score C, none with E, some (9%) A. Ready meals classified with Nutri-Score A were products such as fish dishes, salads, and some stews. Pizza got Cs and Ds. Ready meals with red meat are not differentiated from similar meals without red meat. For example, similar pizzas with and without red meat can get classified with the same Nutri-Score class. Similar were observed for composite meals.
- **Semi-ready meals** (n = 42): Prepared stews and soups from powders could be classified with Nutri-Score A or B. Most products (57%) were classified with Nutri-Score C. Unprepared powder products got E, likely because they are concentrated and therefore has a very high salt content. The latter indicate that Nutri-Score should be calculated on the prepared product.
- **Miscellaneous** (n = 43): Consisted of salted crackers/biscuits (lower salt better Nutri-Score); tortillas or wraps (more fiber/whole grain better Nutri-Score class); other types of meat (rabbit, hare, whale – mostly A, pure meat); and sugar/honey (classified with Nutri-Score E).
- **Oils and soft/liquid margarine** (n = 31*)*: No products were classified with Nutri-Score A or E, but soybean oil, olive oil, rapeseed oil, etc. got Bs, while typical margarines got Cs and a few D.
- **Butterblends and hard margarine** (n = 10): The products were classified with Nutri-Score D or E (40% and 60% respectively). Palm oil got D, while hard margarine and butterblends were classified with E except low-salt products.
- **Butter and hard oils** (n = 8): All products were classified with Nutri-Score E. The butter with the highest total points (31 total points) was high in salt, but still got the same Nutri-Score class regular butter (24 total points) and butter without salt (19 total points). Coconut fat got E (20 total points).
- **Cremes** (n = 21): Cremes were classified as either C (14%) or D (85%). The differences in fat content does not seem to be captured as both high-fat options and low-fat all got Ds, such as full-fat cream with 37% fat (16 total points), cooking cream 18% fat (13 total points), and cooking cream with 10% fat (11 total points). Similar was observed for crème fraiche and sour creams. A very low fat content was needed in the products to achieve a Nutri-Score C.
- **Unsalted nuts** (n = 11): Approximately 82% of nuts in this category were classified with Nutri-Score A. All unsalted nuts got A except for cashew nuts without salt (-3 total points) and macadamia nuts (-3 total points).
- **Salted/coated nuts** (n = 13): No products got Nutri-Score class A. Nuts with salt got B and C, and nuts getting Ds were more processed nuts and mixes, such as chili nuts and nut mixes with dried fruit/chocolate.
- **Seeds** (n = 11): These products were mostly classified with Nutri-Score As (91%) or Bs (9%). Only Psyllium flea seeds that got B, the rest A.
- **Pure water** (n = 3): All receive A, as per the updated Nutri-Score for beverages.
- **Other beverages** (n = 28): Consists of various drinks, such as tea, coffee, carbonated water, flavored water and non-alcoholic beer/wine. All products were scored between -2 to 3 total Nutri-Score points, indicating Nutri-Score B, except two products which were classified with Nutri-Score C (sweet teas).
- **Sugar-sweetened beverages** (n = 44): Most (66%) beverages in this category were classified with Nutri-Score D (27%) or E (66%), likely due to the sugar and energy content. Products classified with Nutri-Score C had a lower sugar and energy content.
- **Artificially sweetened beverages** (n = 21): 95% of products in this category were classified with Nutri-Score C. The product classified with Nutri-Score E, was likely due to the natural sugar content in addition to non-nutritive sweeteners.
- **Fruit and vegetable juices** (n = 39): Most products in this category were classified with Nutri-Score C (48%). Beverages classified with Nutri-Score B in this category seem to be lower in energy. Some juices and smoothies got a Nutri-Score C, but some also got a D. Products classified with Nutri-Score E were products containing non-nutritive and/or had a lower fruit, vegetable and legumes content, or a high sugar and energy content (e.g. grape juice).
- **Milk and dairy beverages** (n = 55): Most (38%) milk and dairy beverages were classified with Nutri-Score B. Skimmed- (0.1% fat) and partly skimmed (0.5-1.0% fat) milk were classified with Nutri-Score B, and total Nutri-Score points of -2 and -3 to 0 total points, respectively. The difference in fat content was partly reflected in the total Nutri-Score points for each product (skimmed: -2 total points; partly skimmed: -1 total points), however one partly skimmed milk with 0.5% fat got a total Nutri-Score points of -3 (indicating a higher nutritional quality than skimmed milk). Whole-fat milks with 3.5% fat were classified with Nutri-Score B (2 total points) and C (3 total points), whole milks with 4% and 4.1% fat were classified with C (4 or 5 total points). Fermented milk-based beverages could be classified with Nutri-Score B or C, but most fermented milks with flavorings were classified with Nutri-Score C. Products classified with Nutri-Score E were typically high in energy (kJ) and sugars.
- **Plant-based beverages** (n = 19): Most products (37%) were classified with Nutri-Score D, but some were also classified with Nutri-Score B (26%) or C (26%). Soy and almond milks were mainly classified with Nutri-Score B, but could also be classified with poorer Nutri-Score classes. The various classification of the plant-based beverages varied according to the different sugar and energy content. In general, Nutri-Score does not capture the difference in calcium content between the beverages.
