# Supplementary material for: How does the updated Nutri-Score discriminate and classify the nutritional quality of foods in a Norwegian setting?
Source: Int J Behav Nutr Phys Act. 2023 Oct 10;20:122. doi: 10.1186/s12966-023-01525-y (PMC10563306; doi:10.1186/s12966-023-01525-y)
Supplement: Supplementary file 9 — Additional file 9. Central and dispersion of points from each component in the Nutri-Score algorithms within food categories. [file 12966_2023_1525_MOESM9_ESM.docx]

**Additional file 9. Central and dispersion of points from each component in the Nutri-Score algorithms within food categories.**

| **Table**. Central and dispersion of points given by each component in the Nutri-Score general foods algorithm for each subcategory of foods. | | | | | | | | | | | | | | |
| --- | --- | --- | --- | --- | --- | --- | --- | --- | --- | --- | --- | --- | --- | --- |
|  | | | | | | | | | | | | | | |
|  | Points from unfavorable components | | | | | | | | Points from favorable components | | | | | |
|  | Energy  (0-10 points) | | Sugars  (0-15 points) | | Saturated fat  (0-10 points) | | Salt  (0-20 points) | | Protein  (0-7 points) | | Fiber  (0-5 points) | | FVL-proportion (0,1,2 or 5 points) | |
|  | Median (IQR) | min – max | Median (IQR) | min – max | Median (IQR) | min – max | Median (IQR) | min – max | Median (IQR) | min – max | Median (IQR) | min – max | Median (IQR) | min – max |
| Fruit, vegetables, legumes (n = 233) | 0 (1) | 0 – 8 | 1 (2) | 0 – 15 | 0 (0) | 0 – 10 | 0 (0) | 0 – 20 | 0 (1) | 0 – 7 | 0 (1) | 0 – 5 | 5 (0) | 0 – 5 |
| Potatoes, potato products (n = 22) | 1 (2) | 0 – 4 | 0 (0) | 0 – 1 | 0 (1) | 0 – 5 | 2 (4) | 0 – 12 | 0 (0) | 0 – 3 | 0 (0) | 0 – 4 | 0 (0) | 0 - 0 |
| Grains, pasta, rice, noodles (n = 45) | 4 (0) | 1 - 5 | 0 (0) | 0 - 1 | 0 (0) | 0 - 3 | 0 (0) | 0 - 19 | 4 (2) | 0 - 6 | 1 (3) | 0 - 5 | 0 (0) | 0 - 0 |
| Flour, flour mixes (n = 38) | 4 (0) | 3 - 7 | 0 (0) | 0 - 14 | 0 (0) | 0 - 10 | 0 (0) | 0 - 14 | 4 (4) | 0 - 7 | 5 (3) | 0 - 5 | 0 (0) | 0 - 0 |
| Breads (n = 110) | 3 (1) | 2 - 6 | 0 (0) | 0 - 5 | 0 (1) | 0 - 5 | 5 (2) | 0 - 10 | 3 (1) | 0 - 7 | 4 (3) | 0 - 5 | 0 (0) | 0 - 0 |
| Breakfast cereals (n = 36) | 4 (0) | 4 - 5 | 3 (4.5) | 0 - 12 | 0 (1) | 0 - 10 | 2 (3) | 0 - 13 | 3 (1) | 2 - 6 | 4 (3) | 0 - 5 | 0 (0) | 0 - 0 |
| Eggs (n = 5) | 1 (0) | 0 - 3 | 0 (0) | 0 - 0 | 2 (0) | 0 - 8 | 1 (0) | 0 - 2 | 5 (0) | 4 - 6 | 0 (0) | 0 - 0 | 0 (0) | 0 - 0 |
| Fish, seafood* (n = 103) | 1 (2) | 0 - 6 | 0 (0) | 0 - 4 | 0 (1) | 0 - 9 | 1 (6) | 0 - 20 | 6 (2) | 2 - 7 | 0 (0) | 0 - 0 | 0 (0) | 0 - 0 |
| Meat - red* (n = 139) | 2 (1) | 1 - 9 | 0 (0) | 0 - 4 | 4 (5) | 0 - 10 | 1 (9) | 0 - 20 | 2 (0) | 1 - 2 | 0 (0) | 0 - 2 | 0 (0) | 0 - 0 |
| Meat - poultry* (n = 40) | 1.5 (1) | 1 - 4 | 0 (0) | 0 - 0 | 2 (2) | 0 - 10 | 1 (7) | 0 - 13 | 7 (2) | 4 - 7 | 0 (0) | 0 - 0 | 0 (0) | 0 - 0 |
| Plant-based meat alternatives* (n = 24) | 2 (0) | 0 - 3 | 0 (0) | 0 - 0 | 0 (1.5) | 0 - 8 | 6 (4) | 0 - 10 | 4.5 (3.5) | 2 - 7 | 2 (3.5) | 0 - 5 | 0 (0) | 0 - 2 |
| Yoghurt and plant-based alternatives (n = 44) | 0.5 (1) | 0 - 2 | 2 (1.5) | 0 - 5 | 1 (2) | 0 - 7 | 0 (0) | 0 - 1 | 1 (1) | 0 - 4 | 0 (0) | 0 - 0 | 0 (0) | 0 - 0 |
| Cheese and plant-based alternatives (n = 84) | 3 (1) | 0 - 5 | 0 (0) | 0 - 13 | 10 (0) | 0 - 10 | 6 (5) | 0 - 20 | 7 (3) | 0 - 7 | 0 (0) | 0 - 0 | 0 (0) | 0 - 0 |
| Sandwich toppings (n = 121) | 2 (2) | 0 - 6 | 0 (8) | 0 - 15 | 1 (3) | 0 - 10 | 6 (11) | 0 - 20 | 2 (2) | 0 - 7 | 0 (0) | 0 - 5 | 0 (0) | 0 - 2 |
| Sauces, dressings (n = 68) | 1 (3) | 0 - 8 | 1 (2.5) | 0 - 15 | 1 (3) | 0 - 10 | 8 (10.5) | 0 - 20 | 0 (1) | 0 - 7 | 0 (0) | 0 - 5 | 0 (0) | 0 - 5 |
| Crisps (n = 16) | 5 (1) | 4 - 7 | 0 (0) | 0 - 4 | 2 (1.5) | 0 - 10 | 9.5 (9) | 3 - 20 | 2.5 (1.5) | 1 - 7 | 2.5 (2.5) | 0 - 5 | 0 (0) | 0 - 0 |
| Chocolate, candy (n = 59) | 5 (12) | 2 - 7 | 15 (2) | 0 - 15 | 10 (8) | 0 - 10 | 0 (1) | 0 - 6 | 2 (2) | 0 - 7 | 0 (1) | 0 - 5 | 0 (0) | 0 - 0 |
| Sweet biscuits/pastries (n = 45) | 5 (1) | 3 - 6 | 6 (4) | 2 - 15 | 5 (8) | 1 - 10 | 3 (2) | 0 - 9 | 2 (1) | 0 - 4 | 0 (1) | 0 - 5 | 0 (0) | 0 - 0 |
| Desserts, cakes, ice cream (n = 92) | 2.5 (3) | 0 - 6 | 6 (7) | 0 - 15 | 2 (6.5) | 0 - 10 | 0 (2) | 0 - 8 | 1 (2) | 0 - 6 | 0 (0) | 0 - 5 | 0 (0) | 0 - 1 |
| Ready meals (n = 59) | 1 (1) | 0 - 5 | 0 (1) | 0 - 3 | 2 (3) | 0 - 10 | 4 (2) | 0 - 8 | 2 (2) | 0 - 6 | 0 (0) | 0 - 5 | 0 (0) | 0 - 2 |
| Semi-ready meals (n = 42) | 1 (3) | 0 - 5 | 0 (0) | 0 - 12 | 0 (1) | 0 - 7 | 4 (3) | 0 - 20 | 1 (4) | 0 - 7 | 0 (0) | 0 - 5 | 0 (0) | 0 - 2 |
| Miscellanous (n = 43) | 3 (1) | 1 - 5 | 0 (2) | 0 - 15 | 0 (1) | 0 - 10 | 3 (7) | 0 - 20 | 3 (2) | 0 - 7 | 1 (3) | 0 - 5 | 0 (0) | 0 - 5 |
| Total (n = 1468) | 2 (3) | 0 - 9 | 0 (2) | 0 - 15 | 1 (4) | 0 - 10 | 2 (6) | 0 - 20 | 2 (3) | 0 - 7 | 0 (1) | 0 - 5 | 0 (0) | 0 - 5 |
| *Excluding typical spreads or cold cuts used as sandwich toppings as they are included in the sandwich toppings category. FVL: fruit, vegetables and legumes; IQR: Interquartile range; | | | | | | | | | | | | | | |

| **Table**. Central and dispersion of points given by each component in the Nutri-Score algorithm for fats/oils and nuts/seeds. | | | | | | | | | | | | | | |
| --- | --- | --- | --- | --- | --- | --- | --- | --- | --- | --- | --- | --- | --- | --- |
|  | Points from unfavorable components | | | | | | | | Points from favorable components | | | | | |
|  | Energy from saturated fat  (0-10 points) | | Sugars  (0-15 points) | | Saturated fat/total fat  (0-10 points) | | Salt  (0-20 points) | | Protein  (0-7 points) | | Fiber  (0-5 points) | | FVL-proportion  (0, 1, 2 or 5 points) | |
|  | Median (IQR) | min – max | Median (IQR) | min – max | Median (IQR) | min – max | Median (IQR) | min – max | Median (IQR) | min – max | Median (IQR) | min – max | Median (IQR) | min – max |
| Fats, oils (n = 70) | 4 (6) | 0 - 10 | 0 (0) | 0 - 2 | 6 (7) | 0 - 10 | 0 (4) | 0 - 14 | 0 (0) | 0 - 3 | 0 (0) | 0 - 0 | 0 (0) | 0 - 5 |
| Nuts, seeds (n = 35) | 1 (1) | 0 - 3 | 1 (1) | 0 - 6 | 1 (2) | 0 - 4 | 0 (4) | 0 - 13 | 7 (1) | 0 - 7 | 5 (0) | 1 - 5 | 0 (0) | 0 - 0 |
| Total (n = 105) | 2 (4) | 0 - 10 | 0 (1) | 0 - 6 | 2 (8) | 0 - 10 | 0 (4) | 0 - 14 | 0 (6) | 0 - 7 | 0 (5) | 0 - 5 | 0 (0) | 0 - 5 |
| FVL: fruit, vegetables and legumes; IQR: Interquartile range. | | | | | | | | | | | | | | |

| **Table**. Central and dispersion of points given by each component in the Nutri-Score algorithm for subcategories of beverages*. | | | | | | | | | | | | | | | | |
| --- | --- | --- | --- | --- | --- | --- | --- | --- | --- | --- | --- | --- | --- | --- | --- | --- |
|  | Points from unfavorable components | | | | | | | | | | Points from favorable components | | | | | |
|  | Energy  (0-10 points) | | Sugars  (0-10 points) | | Saturated fat  (0-10 points) | | Salt  (0-20 points) | | NNS (absence = 0 points / presence = 4 points) | | Protein  (0-7 points) | | Fiber  (0-5 points) | | FVL-proportion  (0, 2, 4 or 6 points) | |
|  | Median (IQR) | min – max | Median (IQR) | min – max | Median (IQR) | min – max | Median (IQR) | min – max | Median (IQR) | min – max | Median (IQR) | min – max | Median (IQR) | min – max | Median (IQR) | min – max |
| Other beverages (n = 28) | 0 (1) | 0 - 6 | 0 (0.5) | 0 - 4 | 0 (0) | 0 - 1 | 0 (0) | 0 - 0 | 0 (0) | 0 - 4 | 0 (0) | 0 - 4 | 0 (0) | 0 - 5 | 0 (0) | 0 - 6 |
| Sugar-sweetened beverages (n = 44) | 3 (1) | 1 - 10 | 9 (3) | 2 - 10 | 0 (0) | 0 - 0 | 0 (0) | 0 - 1 | 0 (0) | 0 - 4 | 0 (0) | 0 - 2 | 0 (0) | 0 - 0 | 0 (0) | 0 - 0 |
| Artificially sweetened beverages (n = 21) | 0 (0) | 0 - 4 | 0 (0) | 0 - 10 | 0 (0) | 0 - 0 | 0 (0) | 0 - 1 | 4 (0) | 0 - 4 | 0 (0) | 0 - 0 | 0 (0) | 0 - 0 | 0 (0) | 0 - 0 |
| Fruit and vegetable-based beverages (n = 39) | 3 (1) | 0 - 6 | 9 (1) | 0 - 10 | 0 (0) | 0 - 1 | 0 (0) | 0 - 2 | 0 (0) | 0 - 4 | 0 (0) | 0 - 0 | 0 (0) | 0 - 0 | 6 (0) | 0 - 6 |
| Milk and dairy (n = 55) | 4 (3) | 2-10 | 3 (5) | 2 - 10 | 0 (0) | 0 - 5 | 0 (0) | 0 - 2 | 0 (0) | 0 - 4 | 7 (1) | 1 - 7 | 0 (0) | 0 – 0 | 0 (0) | 0 - 0 |
| Plant-based milk alternatives (n = 19) | 3 (3) | 1 - 8 | 3 (5) | 0 - 6 | 0 (0) | 0 - 0 | 0 (0) | 0 - 0 | 0 (0) | 0 - 0 | 0 (4) | 0 - 7 | 0 (0) | 0 - 0 | 0 (0) | 0 - 0 |
| Total (n = 206) | 3 (3) | 0 - 10 | 4 (7) | 0 - 10 | 0 (0) | 0 - 5 | 0 (0) | 0 - 2 | 0 (0) | 0 - 4 | 0 (6) | 0 - 7 | 0 (0) | 0 - 5 | 0 (0) | 0 - 6 |
| *Plain water is excluded as it is not calculated but automatically gets Nutri-Score class A. FVL: fruit, vegetables, and legumes; IQR: Interquartile range; NNS: Non-nutritive sweeteners. | | | | | | | | | | | | | | | | |
