# Supplementary material for: How does the updated Nutri-Score discriminate and classify the nutritional quality of foods in a Norwegian setting?
Source: Int J Behav Nutr Phys Act. 2023 Oct 10;20:122. doi: 10.1186/s12966-023-01525-y (PMC10563306; doi:10.1186/s12966-023-01525-y)
Supplement: Supplementary file 10 — Additional file 10. Breads with the bread scale and Nutri-Score. [file 12966_2023_1525_MOESM10_ESM.docx]

**Additional file 10. Breads with the bread scale and Nutri-Score**

**Table.** Breads (n = 71) categorized according to the Bread Scale label and the distribution of Nutri-Score

|  | Nutri-Score class | | | | | Total |
| --- | --- | --- | --- | --- | --- | --- |
| Coarseness | A | B | C | D | E |  |
| 0-25% | 0.0 (0) | 22.7 (5) | 59.1 (13) | 18.2 (4) | 0.0 (0) | 100.0 (22) |
| 25-50% | 20.0 (2) | 50.0 (5) | 30.0 (3) | 0.0 (0) | 0.0 (0) | 100.0 (10) |
| 50-75% | 54.2 (13) | 20.8 (5) | 25.0 (6) | 0.0 (0) | 0.0 (0) | 100.0 (24) |
| 75-100% | 93.3 (14) | 6.7 (1) | 0.0 (0) | 0.0 (0) | 0.0 (0) | 100.0 (15) |
| Total | 40.8 (29) | 22.5 (16) | 31.0 (22) | 5.6 (4) | 0.0 (0) | 100.0 (71) |
| Data shown as percentages (n). | | | | | | |
